# Etiology and Symptoms of Maize Leaf Spot Caused by *Bipolaris* spp. in Sichuan, China

**DOI:** 10.3390/pathogens9030229

**Published:** 2020-03-20

**Authors:** Xiaofang Sun, Xiaobo Qi, Wei Wang, Xuan Liu, Henan Zhao, Cuiping Wu, Xiaoli Chang, Min Zhang, Huabao Chen, Guoshu Gong

**Affiliations:** College of Agronomy, Sichuan Agricultural University, Chengdu 611130, China; sunxiaofang207@163.com (X.S.); xiaoboQi1314@126.com (X.Q.); ww193624@163.com (W.W.); lx665461@163.com (X.L.); zhn1128a@163.com (H.Z.); wucuiping123@163.com (C.W.); xl_changkit@126.com (X.C.); yalanmin@126.com (M.Z.); Chenhuabao12@163.com (H.C.)

**Keywords:** maize leaf spot, *Bipolaris*, identification, symptom, diagnosis

## Abstract

Many species of the genus *Bipolaris* are important plant pathogens and often cause leaf spot, root rot, and seedling blight in an extremely wide range of hosts around the world. In recent years, maize leaf spot caused by *Bipolaris* species has frequently occurred with complex symptoms and is becoming increasingly serious in Sichuan Province of China. To investigate the population diversity of *Bipolaris* spp. and their corresponding symptoms in maize, 747 samples of maize leaf spot were collected from 132 sampling sites in 19 administrative districts of Sichuan Province from 2011 to 2018. Based on morphological characteristics, pathogenicity testing, and phylogenetic analysis of the rDNA internal transcribed spacer (ITS) and glyceraldehyde-3-phosphate dehydrogenase (GAPDH) genes, a total of 1186 *Bipolaris* isolates were identified as *B. maydis*, *B. zeicola*, *B. cynodontis*, *B. oryzae*, *B. setariae,* and *B. saccharicola*, among which *B. maydis* and *B. zeicola* were the dominant pathogenic species, accounting for 57.34% and 42.07% of the isolates, respectively. We found that *B. zeicola* isolates were mainly distributed in high altitude and cool mountainous areas, while *B. maydis* was more widely distributed in Sichuan Province. The typical symptoms caused by the *Bipolaris* species were clearly distinct in maize. The typical symptoms caused by *B. maydis* were elongated strip lesions, or fusiform, elliptical lesions, and those caused by *B. zeicola* were narrow linear lesions. Herein, *B. saccharicola* was first reported on maize and caused subrotund lesions. This study provides useful information for disease diagnosis and management for *Bipolaris* leaf spot in maize.

## 1. Introduction

Maize (*Zea mays* L.) is one of the most important cereal crops around the world. In China, maize is planted on a total of 42.399 million hectares annually, and the annual production reached 259.071 million tons in 2017 [1]. In southwest China, the cultivation area and production of maize are the third largest in China, accounting for approximately 30% and 30%, respectively [2]. However, maize plantings in this region endure complex ecological conditions with altitudes ranging from 200 to 2500 m. In addition, diverse cultivation practices, including straw return and no- or reduced-tillage, are widely performed, resulting in the gradual increase of maize disease over the year [3]. Recently, leaf spot disease characterized by symptoms of elongated, elliptical lesions or small, narrow linear lesions has occurred and increased in incidence yearly, especially among summer-sown maize plants. However, the complexity and diversity of the symptoms make determining the pathogen species based only on the leaf spot symptoms in the field very difficult. Our preliminary diagnosis suggested that species of the *Bipolaris* genus may be the major causal organisms of this kind of maize leaf spot in Sichuan Province. Therefore, in this study, we focused on *Bipolaris* species, and the maize leaf spot caused by *Bipolaris* species was referred to as *Bipolaris* leaf spot of maize.

*Bipolaris* (anamorph of the ascomycetous genus *Cochliobolus*), which has more than 100 species, is an important genus of plant pathogens [4,5,6,7,8]. Most *Bipolaris* species are associated with leaf spot or blight, root rot, ear rot, seedling blight, and other diseases of cultivated and wild gramineous plants [6,7]. Many species of the *Bipolaris* genus are of considerable economic importance, such as *B. oryzae*, *B. maydis* and *B. sorokiniana*, causing devastating diseases in cereal crops [6,7,8]. Southern leaf blight caused by *B. maydis* is an important maize disease worldwide [5,6,7,9,10]. In China, a more than 30% production reduction in maize is likely caused by southern leaf blight in some regions with serious disease occurrence [2]. In addition, *B. zeicola* also causes northern leaf blight, which resulted in a heavy economic loss in the maize belt of the USA in the 1940s [5]. In China, northern leaf blight has become an important factor in maize production in northeastern China and northern China and has also spread towards Southwest China [11,12,13]. In Sichuan, with the changes in cultivation practices and replacement of the main maize varieties, *Bipolaris* leaf spot of maize has tended to increase in recent years [14].

*Bipolaris* species were formerly described as *Helminthosporium.* In several taxonomic refinements, the *Helminthosporium* species were segregated into four genera: *Bipolaris*, *Curvularia*, *Drechslera*, and *Exserohilum* [4,5]. These genera were morphologically similar and known as helminthosporioid fungi. Some species of these sister genera were also reported to cause maize leaf spot and often mixed infections formed under certain circumstances, especially for the genera *Curvularia* and *Exserohilum* [5,6]. Multiple species of *Curvularia*, including *C. lunata*, *C. pallescens*, *C. eragrostidis*, *C. clavata*, *C. intermedia*, *C. inaequalis*, *C. spicifera,* and *C. papendorfii*, were reported to infect leaves, sheaths, and bracts of maize [15,16,17,18]. *Exserohilum turcicum* and *E. rostratum* were also reported to cause serious maize leaf spot [19,20]. Under field conditions, the symptoms of maize leaf spot caused by these species were very similar and diverse. Accurately distinguishing these species based on symptoms alone is problematic. The identification of *Bipolaris* species is typically based on morphological characteristics; however, many species have similar characteristics, and conidial features are sometimes variable depending on isolates and culture conditions [6,8,21,22,23]. Recently, molecular phylogenetic analyses based on rDNA internal transcribed spacer (ITS), glyceraldehyde-3-phosphate dehydrogenase (GAPDH), translation elongation factor 1α (TEF1α), and large subunit of nuclear ribosomal DNA (LSU) made it possible to determine clear phylogenetic positions in the genus *Bipolaris* and its sister genera [6,22,24,25]. Thus, accurate identification of *Bipolaris* species based on morphological characteristics combined with molecular data has become a trend.

Sichuan Province, located in southwest China, is characterized by a mild and cool climate, with abundant rainfall and less sunshine. The maize planting region in Sichuan has an average annual temperature of 16–18 °C, and an average annual rainfall of 1000–1200 mm. However, this kind of climate is relatively favorable for the infection, colonization, reproduction, and dispersal of *Bipolaris* species [12,14]. Moreover, symptoms of these diseases are especially complicated by host variety differences and variations in pathogen virulence. To our knowledge, the occurrence and population structure of *Bipolaris* species associated with maize leaf spot has not been investigated in Sichuan, China. Thus, the objectives of this study were as follows: (i) to identify the *Bipolaris* diversity associated with maize leaf spot in Sichuan based on morphological and phylogenetic analyses; (ii) to ascertain the corresponding symptoms on maize leaves caused by different *Bipolaris* species; and (iii) to verify the pathogenicity and population distribution of the dominant species to provide guidance for disease diagnosis and control.

## 2. Results

### 2.1. Symptom Types of Maize Leaf Spot Caused by Bipolaris Species

**Figure 1:**
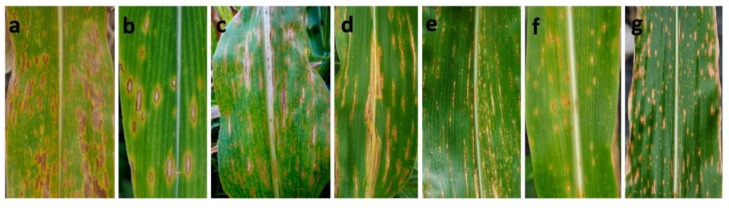
Typical symptoms of *Bipolaris* leaf spot of maize in the field. (**a**,**b**): Type I symptoms included fusiform or elliptical lesions; (**c**,**d**): Type II symptoms were characterized by elongated, nearly long strip lesions restricted by veins; (**e**): Type III symptoms were long, narrow linear lesions; (**f**): Type IV symptoms were nearly circular lesions that were smaller than the Type I lesions; (**g**): Type V symptoms were punctiform or minute necrotic lesions.

A total of 747 symptomatic samples were collected from 132 sampling sites from 2011 to 2018 in 19 administrative districts of Sichuan Province of China. Based on the shape and size of the lesions, the following five typical symptom types were noted in the infected maize leaves collected from the field. Type I (341 samples): fusiform or elliptical lesions, 6–20 mm × 4–11 mm (Figure 1a,b); Type II (135 samples): longitudinally elongated lesions, restricted by veins, developing into long strips of lesions, 5–40 mm × 3–10 mm (Figure 1c,d); Type III (126 samples): long, narrow linear lesions, 3–20 mm × 0.5–2 mm (Figure 1e); Type IV (100 samples): subrotund lesions, which were smaller than the lesions of Type I (Figure 1f); Type V (45 samples): punctiform or minute necrotic lesions (Figure 1g). Type I was the dominant symptom type, accounting for 45.6% of symptoms. In general, more than one symptom type was observed in one sample site or even in the same field.

### 2.2. Morphological Identification of Bipolaris Species Associated with Maize Leaf Spot

**Figure 2:**
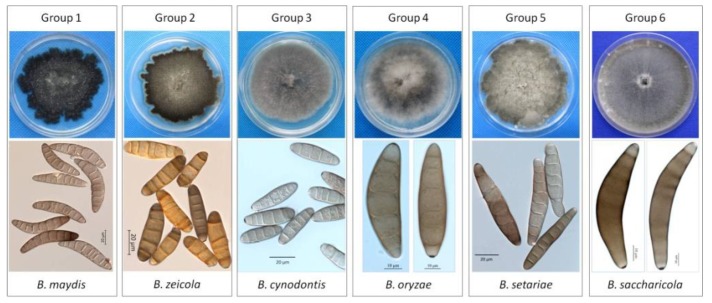
Morphology and cultural characteristics of six *Bipolaris* groups.

A total of 1186 *Bipolaris* single-spore isolates were obtained from all the collected maize leaf spot samples (Table 1 and Figure S1). The 1186 isolates were classified into 6 groups according to their cultural and morphological characteristics. Group 1 included 680 isolates, accounting for 57.34%, fitting the description of *B. maydis*, and Group 2 included 499 isolates, accounting for 42.07%, fitting the description of *B. zeicola*. In addition, Group 3 consisted of 3 isolates matching the description of *B. cynodontis*, and Group 4 had one isolate fitting the description of *B. oryzae*. Two isolates in Group 5 and one isolate in Group 6 fit the description of *B. setariae* and *B. saccharicola*, respectively. Group 1 and Group 2 were the predominant groups. A summary of the morphological data for these *Bipolaris* species in Groups 1-6 is presented in Table 2 and Figure 2.

Colony characteristics (Table 2 and Figure 2): Distinct colony morphology on potato dextrose agar (PDA) was observed for each group after 7 days. The isolates in Group 1 produced dark gray to black colonies with abundant sporulation. The colonies produced by Group 2 isolates were blackish gray with marginally pigmented zones, abundant sporulation and irregular margins. The isolates in Group 3 produced gray to grayish dark colonies with sparse gray aerial mycelia, and the colonies showed a cottony appearance. The colonies from Group 4 exhibited white to slightly gray mycelia with a fluffy cottony appearance. The colonies produced by Group 5 isolates were slightly gray to dark gray, with abundant aerial mycelia. The isolate from Group 6 produced olivaceous gray to olivaceous black, moderate aerial mycelia with a cottony appearance.

Growth rate (Table 2): Isolates from different groups exhibited different growth rates. The maximum mycelial growth was observed in Group 6 (5.8 ± 0.3 mm/day), followed by those from Group 1 (5.7 ± 0.2 mm/day), Group 5 (5.5 ± 0.13 mm/day), Group 3 (5.1 ± 0.11 mm/day), Group 4 (4.8 ± 0.09 mm/day), and Group 2 (4.5 ± 0.19 mm/day).

Conidial morphology (Table 2 and Figure 2): Two conidial types were observed among the six groups based on the following conidia shapes: curved or slightly straight (observed in Groups 1, 4, and 6) and straight or slightly curved (observed in Groups 2, 3, and 5). The conidia sizes of Groups 1, 4, and 6 were similar, but the conidia color produced by the Group 1 isolates was light fawn to dark brown, which was different from those of Groups 4 and 6. The length-width ratio of Group 1 was larger than those of Group 4 and Group 6. Group 4 isolates produced fusiform, obclavate, slightly brown to brown conidia. The conidia produced by Group 6 isolates were fusiform, subhyaline to pale brown or brown, and the conidia of Group 6 were relatively longer and prominently curved. Isolates of Groups 2, 3, and 5 had significant differences in conidia color. The conidia produced by the Group 2 isolates were narrowly elliptical to obclavate, helvus, and isabelline to dark brown. Group 3 produced cylindrical to ellipsoidal, light olivaceous green to brown or golden brown conidia, and the conidia of Group 5 were fusiform or obclavate, and pale brown to dark brown.

### 2.3. Phylogenetic Analysis

A total of 173 isolates from the six morphological groups were subsequently selected for further molecular analyses based on their ITS and GAPDH gene sequences. The amplification success rates of ITS and GAPDH were 92.5% and 86.7%, respectively. The GenBank accession numbers of these *Bipolaris* isolates are listed in Table S1. The referred sequences of helminthosporioid fungi (35 *Bipolaris*, 11 *Curvularia*, 6 *Drechslera,* and 13 *Exserohilum*) and one other species, *Alternaria alternata*, originating from GenBank and used for phylogenetic analysis are listed in Table 3.

#### 2.3.1. Phylogeny Based on the ITS Region

A maximum-parsimony tree was constructed based on the ITS gene region of 160 isolates obtained in this study, 20 reference taxa and an outgroup (*Alternaria alternata*), as shown in Figure S2 (TL = 62 steps, CI = 0.935, RI = 0.994, and RCI = 0.930). There were 465 characteristics in this analysis, including 398 conserved characteristics and 23 parsimony informative characteristics. A total of 160 isolates were clustered into six separate clades corresponding to *B. maydis*, *B. zeicola*, *B. setariae*, *B. saccharicola*, *B. cynodontis* and *B. oryzae*, which was consistent with the morphological analyses.

#### 2.3.2. Phylogeny Based on the GAPDH Region

A maximum-parsimony tree was constructed based on the GAPDH gene region containing 150 isolates obtained in this study, 19 reference taxa and an outgroup (*Alternaria alternata*), as shown in Figure S3 (TL = 129 steps, CI = 0.907, RI = 0.994, and RCI = 0.902). For the 498 characteristics used in the phylogenetic analysis, 385 were conserved, and 57 were parsimony informative. The tree contained six primary clades, including *B. maydis*, *B. zeicola*, *B. setariae*, *B. saccharicola*, *B. cynodontis*, and *B. oryzae*, with their corresponding *Bipolaris* species from GenBank.

#### 2.3.3. Phylogeny Based on the ITS + GAPDH Regions

**Figure 3:**
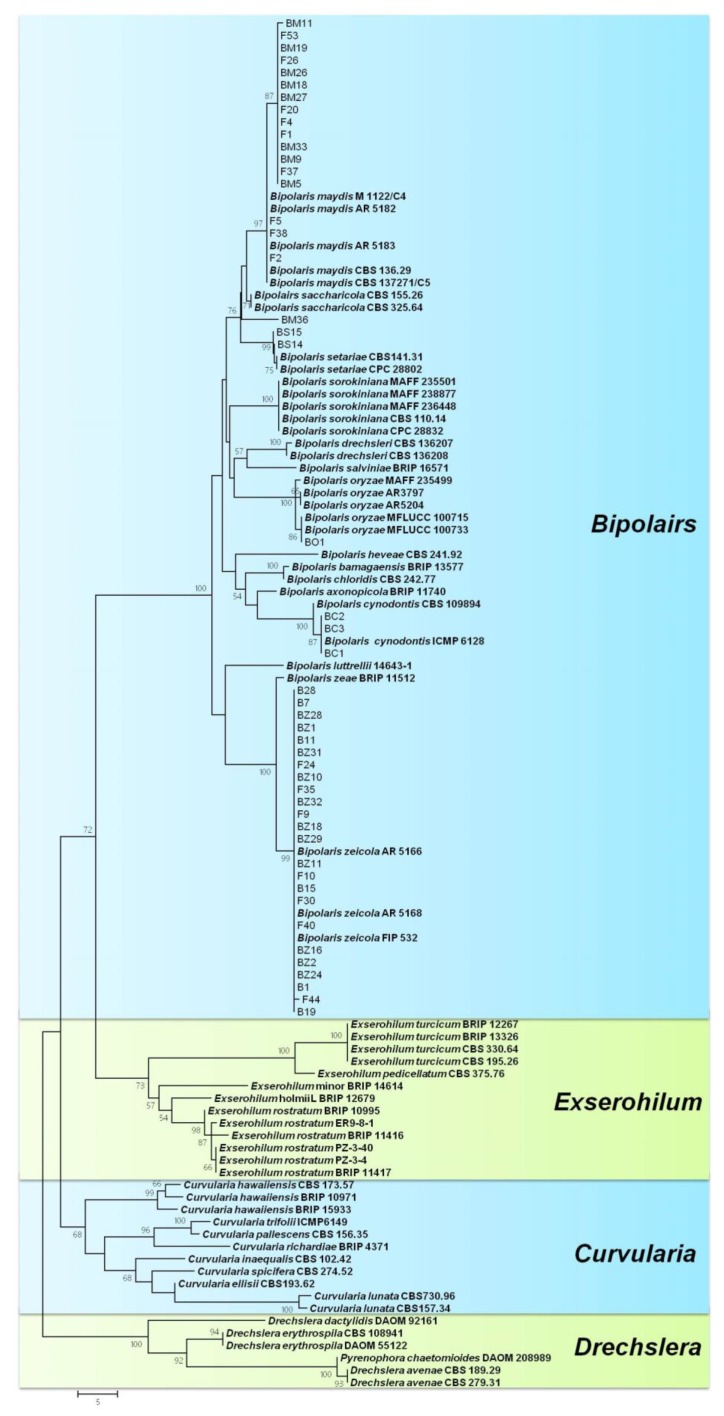
Phylogram generated from parsimony analysis based on combined internal transcribed spacer (ITS) and glyceraldehyde-3-phosphate dehydrogenase (GAPDH) sequenced data of the accepted species of *Bipolaris*, *Curvularia, and Exserohilum*. Numbers on the branching points are ≥50% bootstrap values from a bootstrap test of 1000 replicates. Isolates obtained from NCBI are indicated in bold. The tree was rooted with *Drechslera* species.

To evaluate the phylogenetic relationship among species of *Bipolaris*, multilocus phylogenetic analysis of the combined sequences of ITS and GAPDH gene regions was performed among 81 isolates of *Bipolaris* (48 isolates from this study), 13 isolates of *Exserohilum*, 11 isolates of *Curvularia*, and 6 isolates of *Drechslera* (used as outgroup). The dataset contained 780 characteristics, of which 537 were consistent and 203 were parsimony informative (TL = 298 steps, CI = 0.557, RI = 0.910, and RCI = 0.507). The phylogram was divided into four clades, as shown in Figure 3. Species of *Bipolaris* formed a well-supported clade (100% bootstrap values), clearly separated from other graminicolous helminthosporioid genera, such as *Exserohilum*, *Curvularia*, and *Drechslera*. Similar to the ITS and GAPDH trees, six *Bipolaris* species in this study were clearly separated in this tree and clustered into six distinct clades with their corresponding species from GenBank.

### 2.4. Fungal Species and Corresponding Field Symptom Types

The isolation frequencies of fungal species isolated from the maize leaf spot samples with different symptom types are recorded in Table 4. *B. maydis* and *B. zeicola* could be isolated from all symptom types, and these two species were simultaneously isolated from 111 samples. *B. maydis* were mainly isolated from fusiform, elliptical (Type I) and elongated (Type II) lesions, with 54.4% and 77.8% isolation frequencies, respectively. A total of 432 isolates of *B. maydis* were obtained from Type I and Type II samples. Type I is the most typical symptom caused by *B. maydis* in the field and is highly concerned by growers. It has been learned from investigation that a lower isolation frequency of *B. maydis* from Type I should be attributed to the use of fungicides during the growing season. However, most *B. zeicola* were isolated from long narrow linear lesions (Type III), with 100% isolation frequency, and 235 isolates of *B. zeicola* were obtained. Except for these main types, both *B. maydis* and *B. zeicola* could be obtained from subrotund (Type IV) and punctiform (Type V) samples. *B. saccharicola* and *B. setariae* were isolated from subrotund lesions (Type IV). *B. cynodontis* and *B. oryzae* were isolated from punctiform lesions (Type V). Moreover, a fair number of isolates of the genera *Curvularia*, *Colletotrichum* and *Exserohilum* were isolated along with the *Bipolaris* species in the present study. One hundred fifty-one *Curvularia* isolates were frequently obtained from samples of Type IV and Type V symptoms, but the subrotund lesions caused by *Curvularia* spp. were smaller and more densely distributed than lesions caused by *Bipolaris* species. Ninety-nine isolates of *Colletotrichum* spp. were mainly isolated accompanying *Bipolaris* species from Type I and Type II samples. Eleven isolates of *E. rostratum* were isolated from 4 Type I samples.

### 2.5. Pathogenicity Tests

Seventy-four representative isolates from six identified *Bipolaris* species were used for pathogenicity testing on maize (Table S2). After one week of inoculation, all inoculated leaves developed characteristic lesions, whereas untreated controls had no symptoms. Five kinds of symptoms were observed in the pathogenicity test: elliptical, fusiform, subrotund, narrow linear, and punctiform. Leaves inoculated with *B. maydis* showed small, light brown watery spots at 48 h after inoculation. Subsequently, these small spots developed into subrotund, or elliptical, fusiform lesions in 5–7 days. Some lesions joined together and caused a large area of leaf death. The main symptoms caused by *B. zeicola* were small spots in the first three days, and then, for some isolates, narrow linear lesions appeared and extended to 5–13 mm in length at 7 days after inoculation. The isolate BM36 of *B. saccharicola* caused subrotund spots of approximately 5 mm in diameter on maize leaves at 7 days after inoculation. Leaves inoculated with *B. setariae* isolates showed symptoms of small subrotund spots after 7 days of inoculation. Only small chlorotic punctiform spots appeared on maize leaves after 3 days when inoculated with *B. cynodontis* and *B. oryzae*, but the spots did not extend further over time. The diseased leaves were collected at 10 days after inoculation, and the recovered fungi were consistent with the inoculated species.

**Figure 4:**
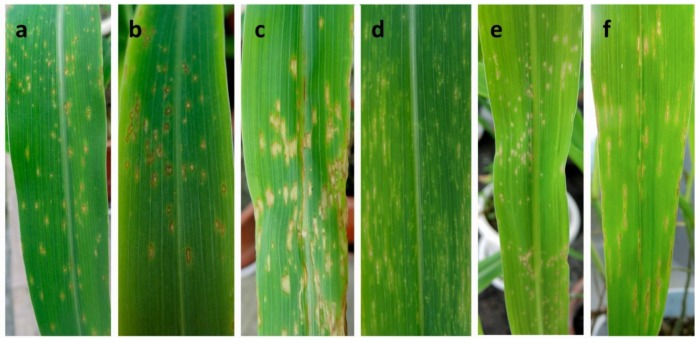
Symptoms in maize leaves after inoculation with the two predominant *Bipolaris* species: *B. maydis* and *B. zeicola.* (**a**–**c**): different symptoms at 7 days after inoculation with *B. maydis*, each representing weak (**a**), moderate (**b**), and high (**c**) virulence; (**d**–**f**): different symptoms at 7 days after inoculation with *B. zeicola*, each representing weak (**d**), moderate (**e**), and high (**f**) virulence.

Virulence differentiation was observed among the predominant species of *B. maydis* and *B. zeicola* with different symptoms (Figure 4). According to the lesion length, isolate virulence was divided into three categories (Table 5). Moderately and weakly virulent isolates of *B. maydis* accounted for 30.6% and 22.2%, respectively, causing elliptical to subrotund lesions of approximately 2–5 mm length (Figure 4a,b). *B. maydis* isolates with high virulence caused larger fusiform, elliptical or subrotund lesions 6–8 mm in length (Figure 4c) and accounted for 47.2% of the total isolates. *B. zeicola* isolates of weak virulence accounted for 45.2% of the total isolates, only showing small punctiform or white pinhole-like lesions (Figure 4d). *B. zeicola* isolates with moderate virulence causing small subrotund spots (Figure 4e) accounted for approximately 32.2% of isolates, while highly virulent *B. zeicola* isolates accounted for 22.6% of isolates and caused conspicuous long narrow linear lesions, which was the main symptom type observed under field conditions (Figure 4f).

## 3. Discussion

Maize leaf spot is a serious fungal disease worldwide [33]. Accurate diagnosis of *Bipolaris* leaf spot of maize and the associated causal agents plays a key role in the effective management of this disease [7]. According to morphological, phylogenetic, and pathogenicity analysis, our study demonstrated that six *Bipolaris* species were associated with maize leaf spot in Sichuan Province, China. Among them, *B. maydis* and *B. zeicola* were the dominant pathogenic species, accounting for 57.42% and 42.07% of the isolates, respectively. *B. cynodontis*, *B. oryzae*, *B. setariae*, and *B. saccharicola* could also cause maize leaf spot but were extremely rare, implying that they might not occupy a major niche for infection on maize.

Previously, some research results on the isolation and identification of pathogens from diseased maize leaves in China have indicated the predominance of *Bipolaris* species [34,35]. Due to the widespread use of susceptible hybrid maize varieties, *Bipolaris* leaf spot has become the major disease in almost all maize production regions, especially in summer-sown maize fields [14]. In addition to the widely distributed *B. maydis* and *B. zeicola*, some other *Bipolaris* species, such as *B. sorokiniana*, have also been reported as harmful pathogens of maize [6,34,36]. *B. sorokiniana*, the causal agent of root rot and leaf spot in wheat, was also recently reported to cause leaf spot in summer-sown maize in North China [36,37]. However, in this study, *B. sorokiniana* was not isolated from the maize samples. We found serious *B. sorokiniana* infection on volunteer wheat seedlings in wheat-maize rotation fields in Sichuan, while isolates of *B. sorokiniana* showed only very weak virulence on maize plants [38]. It was possible that the infection of maize by *B. sorokiniana* required certain strict conditions or that the maize plants used for the pathogenicity test in our study were resistant varieties. The wheat-maize rotation is a traditional planting system in Sichuan, therefore the infection of maize by *B. sorokiniana* is still of great importance. In this study, *B. saccharicola*, *B. setariae*, *B. cynodontis*, and *B. oryzae* were also pathogenic species on maize. *B. saccharicola* is a novel species isolated from *Saccharum officinarum* [22]. This work is the first report of maize leaf spot caused by *B. saccharicola*. Both *B. setariae* and *B. cynodontis* have been reported on maize; however, the former is mainly distributed in Canada, India, and Pakistan [4], and the latter is distributed in Australia and the United States [4,6]. To our knowledge, this is also the first report of *B. setariae* and *B. cynodontis* as causal agents of maize leaf spot in China. Studies by Amorio and Cumagun suggested that in the rice-maize cropping system, *B. oryzae* was not a potential source of inoculum for leaf spot of maize, and likewise, *B. maydis* was not a potential source of inoculum for brown spot of rice [39]. However, *B. oryzae* shows considerable genetic variation within the species; thus, several biotypes and pathotypes may exist within the species [40,41]. The potential threat posed by *B. oryzae* to maize production cannot be underestimated. Since *Bipolaris* species can persist on crop debris for a long time after harvest, this might increase the survival and spread of these pathogens to some extent. Thus, certain environmental conditions may be especially conducive to the interaction of *Bipolaris* species among different hosts. These findings indicate that long-term monitoring of the composition of *Bipolaris* species causing maize leaf spot is still needed.

Comparing the field symptoms with the symptoms shown in the pathogenicity test, the results showed that different *Bipolaris* species caused unique symptom types. Isolates of *B. zeicola* were mainly isolated from type III field symptoms. In the pathogenicity tests, the narrow linear lesions were only observed after inoculation with the isolates of *B. zeicola* obtained in this study. In Yunnan and Shaanxi Provinces of China, *B. zeicola* isolates were also reported to produce long, narrow linear lesions [12,42,43]. Therefore, based on these results, long narrow linear lesions are the typical symptom caused by *B. zeicola.* Isolates of *B. maydis* were mainly isolated from Type I and Type II symptoms. In the pathogenicity tests, the symptoms caused by *B. maydis* isolates were mostly fusiform and elliptical (Type I), and in the late stages, the lesions joined together, causing similar elongated lesions. Therefore, elongated, fusiform and elliptical lesions were the typical symptoms caused by *B. maydis*, which was consistent with the results of previous studies [34,35,36]. Moreover, some subrotund or punctiform lesions were also found to be caused by *B. maydis* or *B. zeicola* isolates, which increased the difficulty of disease diagnosis. *B. saccharicola* and *B. setariae* were isolated from maize leaves with subrotund lesions (Type IV) and caused subrotund lesions in the pathogenicity tests. *B. cynodontis* and *B. oryzae* were isolated from punctiform lesions (Type V) and caused punctiform lesions in the pathogenicity tests. In addition, we also observed a regional difference in the geographic distribution of these two dominant species in Sichuan Province. *B. zeicola* was mainly isolated from Ya’an City, Luding County of Ganzi Prefecture, Xichang City of Liangshan Prefecture, and Beichuan County of Mianyang City, where maize plants were usually planted at high altitude and in cool climates. The samples collected in these regions were almost all Type III. Compared to *B. zeicola*, *B. maydis* had a more extensive distribution. Except for Liangshan Prefecture, *B. maydis* was isolated from each sampling region. Although there were some situations in which the infection of *Curvularia* spp., *Colletotrichum* spp. and *Exserohilum* spp. would further increase the difficulty of disease diagnosis, we can distinguish these species by their particular symptom and distribution characteristics.

Previous studies have shown that the virulence of *B. maydis* and *B. zeicola* isolates is diversified and that these species have different pathotypes [13,42,44,45]. It was reported that race 3 of *B. zeicola* might be a mountain ecotype, favoring high humidity and cool temperature in mountain areas at high elevations [42,46,47,48,49]. *B. zeicola* isolates from Yunnan Province of China, collected from approximately 550 m to 2535 m, were mostly identified as race 3, producing long, narrow linear lesions [42]. Zhang et al. reported that narrow linear lesions on the leaves of mature maize plants were caused by race 3 of *B. zeicola* in Shaanxi Province of China [43]. Therefore, considering these results and the lesion types of our maize samples, most isolates of *B. zeicola* in Sichuan Province may belong to race 3. *B. maydis* is generally subdivided into four races, namely, O, T, C, and S, and among them, race O is the predominant pathogen of maize in China and the disease characterized by the appearance of elongated or fusiform lesions on the leaves of susceptible plants [44,45,50,51]. We inferred that isolates of *B. maydis* in Sichuan may mostly belong to race O. Our pathogenicity tests demonstrated that virulence differentiation existed among isolates of *B. maydis* and *B. zeicola*, which was basically consistent with the findings of previous studies. It is necessary to focus on high virulence groups and establish early-warning lines to avoid huge maize losses. Although most isolates of *B. zeicola* were moderately or weakly virulent in the pathogenicity tests in this study, the maize leaf spot diseases caused by *B. zeicola* were relatively serious in some mountainous locations. Perhaps the virulence testing conditions were somewhat different from the real environmental conditions, and were not suitable for *B. zeicola* infection. Therefore, in future work, we suggest simulating the pathogenicity of *B. zeicola* in cold areas at high altitudes. As a result of the race diversity of *B. maydis* and *B. zeicola*, the disease symptoms on maize are very complicated, and thus, further studies should be devoted to race detection and virulence determination of *Bipolaris* spp. and to the elucidation of the mechanisms of pathogen differentiation.

In conclusion, to explore the species diversity of *Bipolaris* and the corresponding symptoms, a continuous investigation of maize leaf spot was carried out in Sichuan, China. We verified that *B. maydis*, *B. zeicola*, *B. cynodontis*, *B. oryzae*, *B. setariae*, and *B. saccharicola* were able to cause maize leaf spot, while *B. maydis* and *B. zeicola* were the main causal agents of *Bipolaris* leaf spot of maize. These two dominant species were able to cause mixed infections but were distinguished by different symptom characteristics. *B. maydis* was distributed throughout Sichuan Province and mainly caused elongated, fusiform, and elliptical lesions on maize leaves. *B. zeicola* was mainly distributed in cool mountain areas at a high elevation and caused long, narrow linear lesions. These findings contribute greatly to the understanding of the pathogens causing *Bipolaris* maize leaf spot disease.

## 4. Materials and Methods 

### 4.1. Sample Collection and Isolation

From 2011 to 2018, more than 700 symptomatic samples of maize leaves with visible punctiform, linear, fusiform, elliptical, or irregular lesions (at least five maize leaves for each sample) were collected from 132 sampling sites in 19 administrative districts of Sichuan Province during the maize growing season. All sampling sites were distributed in different villages or fields of different towns. Some sites yielded more than one sample, but the samples always originated from different fields.

Isolations were made from one to three lesions per leaf. Tissues were removed from the margins of single lesions, surface-sterilized in 75% ethanol for 30 s and 1% NaClO for 30 s, and rinsed thrice in sterile distilled water. The pieces were placed on sterile potato dextrose agar (PDA) plates containing streptomycin at a concentration of 30 μg·mL^−1^. All PDA plates were incubated at 25 °C in the dark for 5 days. Single-spore cultures were prepared from all fungal colonies that displayed the characteristics of helminthosporioid fungi following the method described by Gong et al. [52]. Conidia were first removed directly from cultured colonies using a sterilized acupuncture needle and dispersed on a plug of 2% water agar on a glass slide. A single spore was picked and placed on another water agar plug under a microscope. Finally, a water agar plug containing only one single spore was transferred to a new PDA plate and cultured at 25 °C until a clear colony was obtained. One isolate per lesion was obtained. Pure cultures were maintained on PDA slants at 4 °C for short-term storage and in 25% glycerol at −80 °C for long-term storage.

### 4.2. Morphological Analysis

All isolates were cultured on PDA media at 25 °C in the dark for 5–7 days. Colony diameter was measured each day from two perpendicular cross-sections until the plates were fully covered. The daily growth rate was calculated based on the values from three replicates. For observation of conidia and conidiophores, sterile cellophane (approximately 2 cm × 2 cm sizes) was placed on a water agar plate, and then mycelial plugs (5 mm diameter) were placed on one end of the cellophane and incubated at 25 °C. After 5–7 days, the cellophane was gently removed with a tweezer and placed on a glass slide for observation under a compound microscope (Axio Imager Z2, Carl Zeiss, Germany). The sizes and shapes of 50 conidia were measured for each isolate. The isolates were tentatively identified based on a comparison with morphological characteristics of colonies and conidia reported in previous taxonomic studies [5,6,22,53].

### 4.3. DNA Extraction and Sequence Amplification

Cultures were grown for 5–7 days on PDA at 25 °C. Mycelia were scraped from the colony surface using a sterile medicine spoon. Genomic DNA of each isolate was extracted using the Ezup Column Fungi Genomic DNA Purification Kit (Sangon Biotech, Shanghai, China) according to the manufacturer’s instructions. DNA concentration and quality were estimated using a Thermo Scientific NanoDrop™ 2000 Spectrophotometer (Thermo Fisher Scientific, Wilmington, Delaware, USA). Then, DNA was diluted to a final concentration of 30 ng·μL^−1^.

The ITS and GAPDH gene sequences of the representative isolates were amplified and sequenced. PCR amplification was conducted with the primer pair ITS1/ITS4 (5’-TCCGTAGGTGAACCTGCGG-3′ and ITS4: 5’-GCTGCGTTCTTCATCGATGC-3′) for the ITS region [54] and GPD1/GPD2: (5′-CAACGGCTTCGGTCGCATTG-3′ and GPD2: 5′-GCCAAGCAGTTGGTTGTGC-3′) for the GAPDH gene [24]. PCR reactions were performed in a final volume of 30 μL containing 1 μL of genomic DNA (30 ng·μL^−1^), 1 μL of each primer (10 μM), 12 μL of ddH_2_O, and 15 μL of 2 × *Taq* PCR MasterMix (Tiangen Biotech, Beijing, China). The ITS region was amplified at 94 °C for 5 min, followed by 30 cycles of 94 °C for 45 s, 58 °C for 45 s, and 72 °C for 2 min with a final extension of 72 °C for 10 min. For the GAPDH region, the amplification program included an initial denaturation step at 96 °C for 2 min, followed by 30 cycles of 1 min at 96 °C, 1 min at 52 °C and 45 s at 72 °C with a final extension of 10 min at 72 °C. The PCR products were analyzed by electrophoresis in 1% agarose gels. DNA sequencing was performed by Sangon Biotech Co. Ltd. (Chengdu, China).

### 4.4. Phylogenetic Analysis

The obtained sequences were compared by BLASTn on the NCBI database, and then reference nucleotide sequences were downloaded from NCBI. All sequences were aligned with Clustal X v2.0, and their characteristics were weighted equally. Maximum-parsimony trees were constructed with MEGA 6.0 based on the Kimura 2-parameter model [55]. The bootstrap values provided on the phylogenetic dendrogram were generated with 1000 replicates, and alignment gaps were excluded. All sequence data generated in this study were deposited in GenBank, and their accession numbers were obtained. The tree length (TL), consistency index (CI), retention index (RI), and rescaled consistency index (RCI) were also calculated.

### 4.5. Pathogenicity Tests

To identify whether all isolated *Bipolaris* species were pathogenic to maize, a total of 74 representative isolates, including 36 isolates of *B. maydis*, 31 isolates of *B. zeicola* and all isolates of the other four less common species, were selected for pathogenicity tests. Seeds of the susceptible maize variety Zhenghong 505 were grown in plastic pots (12 cm in height, 13 cm in diameter of the top) at 23 °C in a greenhouse. Before inoculation, isolates were grown on PDA plates at 27 °C for 5 to 7 days until adequately sporulating. Then, conidia were suspended in sterile water with 0.2% Tween 20 and adjusted to a concentration of 1 × 10^5^ conidia per mL. For each maize plant, 5 mL of conidial suspension was evenly sprayed using a handheld sprayer when plants were at the five- to six-leaf stage [56]. The leaves of control plants were sprayed with sterile distilled water. Six replicates for each isolate and an equal number of inoculated control plants were included. After inoculation, maize plants were covered with plastic film for moisture retention for 24 h at 25 °C in a greenhouse, and then the plastic film was removed. The incubation period was recorded. Lesion length and type were recorded on the third and fourth leaves at 7 days after inoculation. The experiment was repeated three times.

Data from three repetitions of the experiment were combined and subjected to analysis of variance (ANOVA). The presence of lesions and isolate virulence were evaluated by lesion length at 7 days post inoculation as previously described by Kong et al. [50], where high virulence corresponded to lesion length greater than or equal to 6 mm; moderate virulence to lesion length greater than or equal to 4 mm and less than 6 mm; and weak virulence to lesion length less than 4 mm. The organisms were reisolated from the diseased inoculation sites at 10 days after inoculation and identified as the inoculated isolates based on the method mentioned above for the identification of *Bipolaris* species.

## Figures and Tables

**Table 1:** Isolate number of *Bipolaris* species from maize in Sichuan Province, China.

Sampling Regions	No. of Sampling Sites	*B. maydis*	*B. zeicola*	*B. cynodontis*	*B. oryzae*	*B. setariae*	*B. saccharicola*
Chengdu	23	214	97	0	0	2	0
Deyang	12	82	144	3	0	0	0
Meishan	11	94	52	0	0	0	1
Ya’an	13	35	83	0	0	0	0
Mianyang	15	68	23	0	0	0	0
Neijiang	6	36	40	0	1	0	0
Nanchong	6	36	21	0	0	0	0
Leshan	12	32	6	0	0	0	0
Yibin	3	21	3	0	0	0	0
Guang’an	5	17	5	0	0	0	0
Luzhou	3	11	9	0	0	0	0
Guangyuan	7	9	7	0	0	0	0
Suining	4	7	0	0	0	0	0
Ziyang	2	5	1	0	0	0	0
Bazhong	2	6	0	0	0	0	0
Liangshan	2	0	5	0	0	0	0
Ganzi	1	2	3	0	0	0	0
Zigong	2	3	0	0	0	0	0
Dazhou	3	2	0	0	0	0	0
Total	132	680	499	3	1	2	1

**Table 2:** Summary of morphological data for *Bipolaris* isolates.

Group	Species	Colony Characterization	Growth Rate (mm/day)	Conidia			
				Average Length (μm)	Average Width (μm)	Length-Width Ratio	Shape
1(680) †	*B. maydis*	dark grey to black appearance	5.7 ± 0.20a *	93.5 ± 4.0a (52–126)	13.9 ± 0.35b (10–17)	6.6 ± 0.14a (5.6–7.6)	fusiform, slightly curved, light fawn to dark brown, 7–11 distoseptate, hilum distinct, 3–5 μm
2(499)	*B. zeicola*	blackish grey, entire or irrengular margin	4.5 ± 0.19d	64.9 ± 3.1c (42–92)	14.4 ± 0.57b (9–21)	4.5 ± 0.07c (3.8–5.0)	slightly curved or straight, narrow elliptic to obclavate, helvus, isabelline to dark brown, 6–11 distoseptate, hilum inconspicuous
3(3)	*B. cynodontis*	grey to greyish black	5.1 ± 0.11bc	38.2 ± 0.7d (30–45)	11.3 ± 0.15c (10–13)	3.4 ± 0.06d (2.8–4.3)	slightly curved or straight, cylindrical to elliptic, light olivaceous green to brown or golden brown, 3–9 distoseptate, hilum inconspicuous
4(1)	*B. oryzae*	white to slight grey, fluffy cottony	4.8 ± 0.09cd	93.0 ± 4.6a (54–129)	15.9 ± 0.54a (10–21)	5.8 ± 0.12b (4.8–6.8)	curved, rarely straight, fusiform, obclavate, slightly brown to brown, 6–12 distoseptate, hilum slightly protruding
5(2)	*B. setariae*	slight grey to dark grey, with abundant aerial mycelia	5.5 ± 0.13ab	80.3 ± 2.7b (41–120)	13.4 ± 0.33b (9–18)	6.0 ± 0.19b (3.8–9.4)	straight or slightly curved, fusiform or obclavate, pale brown to dark brown, 5–10 distoseptate, hilum inconspicuous or slightly protruding
6(1)	*B. saccharicola*	olivaceous grey to olivaceous black, moderate aerial mycelium giving a cottony appearance	5.8 ± 0.30a	102.4 ± 3.4a (72–138)	17.2 ± 0.69a (10–24)	6.2 ± 0.29ab (4.4–10.8)	curved, rarely straight, fusiform, subhyaline to pale brown or brown, 5–11 distoseptate, hila inconspicuous, brown, slightly protuberant

Note: † The numbers shown in parentheses represent the number of isolates in each group. * The mean difference is significant at the 0.05 level; a–d: the values with the same letter in a column do not significantly differ according to Duncan’s multiple range test.

**Table 3:** Details of the isolates used in this study, including the hosts, locations, and GenBank accession numbers of the generated sequences.

Species	Strain No.^1^	Host	Location Country	GenBank Accession Numbers^2^		References
				ITS	GAPDH	
*Alternaria alternata*	EGS 34.0160	*Arachis hypogaea*	India	AF071346	AF081400	[24]
*Bipolaris axonopicola*	BRIP 11740	*Axonopus fissifolius*	Australia	KX452443	KX452409	[23,26]
*B. bamagaensis*	BRIP 13577	*Brachiaria subquadripara*	Australia	KX452445	KX452411	[23,26]
*B. chloridis*	CBS 242.77	*Chloris gayana*	Australia	JN192372	JN600961	[5,6]
*B. cynodontis*	ICMP 6128	*Cynodon dactylon*	New Zealand	JX256412	JX276427	[25]
*B. cynodontis*	CBS 109894	*Cynodon dactylon*	Hungary	KJ909767	KM034838	[6]
*B. drechsleri*	CBS 136207	*Microstegium vimineum*	USA	KF500530	KF500533	[6]
*B. drechsleri*	CBS 136208	*Microstegium vimineum*	USA	KF500532	KF500535	[6]
*B. heveae*	CBS 241.92	*Hevea* sp.	Nigeria	KJ909763	KM034843	[6,23]
*B. luttrellii*	14643-1	*Dactyloctenium aegyptium*	Australia	AF071350	AF081402	[6,24]
*B. maydis*	CBS 137271/C5	*Zea mays*	USA	AF071325	KM034846	[6,24]
*B. maydis*	AR 5182	*Sorghum bicolor*	Japan	KM230388	KM034844	[6]
*B. maydis*	AR 5183	*Sorghum bicolor*	Japan	KM230390	KM034848	[6]
*B. maydis*	M 1122/C4	*Zea mays*	USA	KM230389	KM034847	[6]
*B. maydis*	CBS 136.29	*Zea mays*	Japan	KJ909769	KM034845	[6]
*B. oryzae*	MFLUCC 100715	*Oryza sativa*	Thailand	JX256416	JX276430	[25]
*B. oryzae*	MFLUCC 100733	*Oryza sativa*	Thailand	JX256417	KM042898	[25]
*B. oryzae*	MAFF 235499	*Oryza sativa*	Japan	KJ922383	KM042897	[6]
*B. oryzae*	AR3797	*Panicum virgatum*	USA	KM230392	KM042894	[6]
*B. oryzae*	AR5204	*Panicum virgatum*	USA	KM230393	KM042895	[6]
*B. saccharicola*	CBS 155.26	*Saccharum officinarum*	—^3^	KY905674	KY905686	[22]
*B. saccharicola*	CBS 325.64	*Saccharum officinarum*	—	KY905675	KY905687	[22]
*B. salviniae*	BRIP 16571	*Salvinia auriculata*	Brazil	KJ415535	KJ415411	[23,26]
*B. setariae*	CBS141.31	—	—	EF452444	EF513206	[22,27]
*B. setariae*	CBSHN01	Cassava	China	GU290228	—	[28]
*B. setariae*	CPC 28802	*Imperata cylindrica*	Thailand	MF490811	MF490833	[23,29]
*B. setariae*	UTHSC 05-3211	Human	USA	HE792921	—	[21]
*B. sorokiniana*	MAFF 236448	*Zea mays*	Japan	KJ909792	KM034826	[6]
*B. sorokiniana*	MAFF 235501	*Zea mays*	Japan	KJ909791	KM034825	[6]
*B. sorokiniana*	MAFF 238877	*Hordeum vulgare*	Japan	KJ909790	KM034824	[6]
*B. sorokiniana*	CBS 110.14	*Hordeum* sp.	USA	KJ922381	KM034822	[6,23]
*B. sorokiniana*	CPC 28832	*Triticum aestivum*	Thailand	MF490812	MF490834	[23,29]
*B. zeae*	BRIP 11512	*Zea mays*	Australia	KJ415538	KJ415408	[23,30]
*B. zeicola*	AR5166	*Sorghum* sp.	USA	KJ909788	KM034813	[6]
*B. zeicola*	AR 5168	*Sorghum* sp.	USA	KM230397	KM034814	[6]
*B. zeicola*	FIP 532	*Zea mays*	USA	KM230398	KM034815	[6]
*Curvularia ellisii*	CBS193.62	Air	Pakistan	JN192375	JN600963	[5]
*C. hawaiiensis*	CBS 173.57	*Oryza sativa*	Hawaii	JN601029	JN600966	[5]
*C. hawaiiensis*	BRIP 10971	*Chloris gayana*	Australia	JN601030	JN600967	[5]
*C. hawaiiensis*	BRIP 15933	*Chloris gayana*	Australia	JN601028	JN600965	[5,6,8]
*C. inaequalis*	CBS 102.42	Sand dune soil	France	KJ922375	KM061787	[6]
*C. lunata*	CBS730.96	Human lung biopsy	USA	JX256429	JX276441	[25]
*C. lunata*	CBS157.34	Unknown	Indonesia	JX256430	JX276442	[25]
*C. pallescens*	CBS 156.35	Air	Java	KJ922380	KM083606	[6]
*C. richardiae*	BRIP 4371	*Richardia brasiliensis*	Australia	KJ415555	KJ415391	[30]
*C. spicifera*	CBS 274.52	Soil	Spain	JN192387	JN600979	[5,6]
*C. trifolii*	ICMP6149	*Setaria glauca*	New Zealand	JX256434	JX276457	[25]
*Drechslera avenae*	CBS 189.29	—	—	AY004795	AY004827	[8,31]
*D. avenae*	CBS 279.31	—	—	AY004796	AY004828	[8,31]
*D. avenae (Pyrenophora chaetomioides)*	DAOM 208989	—	—	AF081445	AF081371	[8]
*D. dactylidis*	DAOM 92161	—	—	AY004781	AY004812	[8,31]
*D. erythrospila*	CBS 108941	—	—	AY004782	AY004813	[8,31]
*D. erythrospila*	DAOM 55122	—	—	AY004783	AY004814	[8,31]
*Exserohilum holmii*	BRIP 12679	*Dactyloctenium radulnas*	Australia	LT837846	LT882542	[32]
*E. minor*	BRIP 14614	*Dactyloctenium aegyptium*	Australia	LT837468	LT715885	[32]
*E. pedicellatum*	CBS 375.76	*Oryza sativa*	Turkey	KT265259	LT715879	[32]
*E. rostratum*	PZ-3-4	*Zea mays*	China	MG780267	MK558815	In this study
*E. rostratum*	PZ-3-40	*Zea mays*	China	MG780268	MK558816	In this study
*E. rostratum*	ER9-8-1	*Zea mays*	China	MG780269	MK558817	In this study
*E. rostratum*	BRIP 10995	*Zea mays*	Australia	LT837823	LT882566	[32]
*E. rostratum*	BRIP 11416	*Zea mays*	Australia	LT837466	LT883543	[32]
*E. rostratum*	BRIP 11417	*Zea mays*	Australia	LT837836	LT882553	[32]
*E. turcicum*	BRIP 12267	*Sorghum bicolor*	Australia	LT837482	LT883553	[32]
*E. turcicum*	BRIP 13326	*Sorghum sudanense*	Australia	LT837480	LT883551	[32]
*E. turcicum*	CBS 195.26	*Zea mays*	Indonesia	LT837485	LT882583	[32]
*E. turcicum*	CBS 330.64	*Zea mays*	USA	LT837484	LT715874	[32]

Note: ^1^ AR, FIP: Isolates housed in Systematic Mycology and Microbiology Laboratory, United States Department of Agriculture, Agricultural Research Service, Beltsville, Maryland; BRIP: Plant Pathology Herbarium, Department of Primary Industries, Queensland, Australia; CBS: CBS-KNAW Fungal Biodiversity Centre, Utrecht, The Netherlands; DAOM: Plant Research Institute, Department of Agriculture (Mycology), Ottawa, Canada; CPC: working collection of P.W. Crous, housed at the Westerdijk Fungal Biodiversity Institute, Utrecht, the Netherlands; EGS: Collection of E. G. Simmons; ICMP: International Collection of Microorganisms from Plants, Landcare Research, Private Bag 92170, Auckland, New Zealand; MAFF: Ministry of Agriculture, Forestry and Fisheries, Tsukuba, Ibaraki, Japan; MFLUCC: Mae Fah Luang University Culture Collection, Chiang Rai, Thailand. ^2^ ITS: internal transcribed spacers and intervening 5.8S nrDNA; GAPDH: partial glyceraldehyde-3-phosphate dehydrogenase gene. ^3^—, not available.

**Table 4:** The isolation frequencies of fungal species isolated from the maize leaf spot samples with different field symptom types.

Symptom Types	Number of Samples	*Bipolaris maydis*	*B. zeicola*	*B. cynodontis*	*B. oryzae*	*B. setariae*	*B. saccharicola*	*Curvularia* spp.	*Colletotrichum* spp.	*Exserohilum rostratum*
Type I: Fusiform or elliptic	341	54.3%	9.97%	0	0	0	0	0	13.2%	1.17%
Type II: Elongate	135	77.8%	18.5%	0	0	0	0	8.89%	22.2%	0
Type III: Narrow linear	126	27.0%	100%	0	0	0	0	15.1%	0	0
Type IV: Subrotund	100	50.0%	24.0%	0	0	1.00%	1.00%	28.0%	3.00%	0
Type V: Punctiform or necrotic	45	26.7%	22.2%	2.22%	2.22%	0	0	46.7%	24.4%	0
Total	747	51.7%	29.3%	0.13%	0.13%	0.13%	0.13%	10.7%	11.9%	0.54%

Note: The isolation frequency is equal to the number of samples with fungal species isolated/total number of samples.

**Table 5:** Virulence differentiation of the two preponderant *Bipolaris* species.

Species	Virulence Level	No. of Isolate	Incubation Period (h)	Average Lesion Length (mm)
*B. maydis*	High virulence	17	24	6.5 ± 0.36b (6–8)
	Moderate virulence	11	24	4.8 ± 0.34c (4.3–5.2)
	Weak virulence	8	48	3.1 ± 0.38e (2.7–3.8)
*B. zeicola*	High virulence	7	24	7.6 ± 0.99a * (6.2–8.9)
	Moderate virulence	10	48	4.3 ± 0.30d (4–5.1)
	Weak virulence	14	72	2.5 ± 0.36f (1.8–2.9)

Note: * The mean difference is significant at the 0.05 level; a–f: the values with the same letter in a column do not significantly differ according to Duncan’s multiple range test.

## Supplementary Materials

The following are available online at https://www.mdpi.com/2076-0817/9/3/229/s1, Figure S1: Map of the *Bipolaris* isolates distribution in the nineteen administrative districts of Sichuan Province, Figure S2: Consensus maximum-parsimony tree based on the ITS gene sequences from 161 *Bipolaris* isolates, Figure S3: Consensus maximum-parsimony tree based on the partial GAPDH gene sequences from 149 *Bipolaris* isolates, Table S1: GenBank accession numbers of *Bipolaris* isolates obtained in this study, Table S2: Symptoms and the virulence levels of *Bipolaris* isolates on maize.
