# Supplementary material for: Etiology and Symptoms of Maize Leaf Spot Caused by Bipolaris spp. in Sichuan, China
Source: Pathogens. 2020 Mar 20;9(3):229. doi: 10.3390/pathogens9030229 (PMC7157660; doi:10.3390/pathogens9030229)
Supplement: Supplementary file 1 [file pathogens-09-00229-s001.zip › Supplementary files/Introduction of Figure S1- S3.docx]

# Figure S1. Map of the *Bipolaris* isolates distribution in the nineteen administrative districts of Sichuan Province. N: isolate number.

# Figure S2. Consensus maximum-parsimony tree based on the ITS gene sequences from 161 *Bipolaris* isolates. Numbers on the branching points are ≥50% bootstrap values from a bootstrap test of 1,000 replicates. Isolates obtained from NCBI are indicated in bold. *Alternaria alternata* (AF071346) is an outgroup.

# Figure S3. Consensus maximum-parsimony tree based on the partial GAPDH gene sequences from 149 *Bipolaris* isolates. Numbers on the branching points are ≥50% bootstrap values from a bootstrap test of 1,000 replicates. Isolates obtained from NCBI are indicated in bold. *Alternaria alternata* (AF081400) is an outgroup.
