# Supplementary material for: Etiology and Symptoms of Maize Leaf Spot Caused by Bipolaris spp. in Sichuan, China
Source: Pathogens. 2020 Mar 20;9(3):229. doi: 10.3390/pathogens9030229 (PMC7157660; doi:10.3390/pathogens9030229)
Supplement: Supplementary file 1 [file pathogens-09-00229-s001.zip › Supplementary files/Table S1. GenBank accession numbers of Bipolaris isolates obtained in this study.docx]

**Table S1.** GenBank accession numbers of *Bipolaris* isolates obtained in this study.

| **Species** | **Isolates No.** | **Group** | **Host** | **Year** | **Location** | **Sampling coordinates** | **Altitude** | **GenBank accession numbers** | |
| --- | --- | --- | --- | --- | --- | --- | --- | --- | --- |
|  |  |  |  |  |  |  |  | **ITS** | **GAPDH** |
| *Bipolaris maydis* | T7 | Group 1 | Maize | 2011 | Ya’an, Sichuan | 29° 59' 59" N, 102° 50' 59" E | 665 | KP211432 | — |
| *B. maydis* | T19 | Group 1 | Maize | 2011 | Ya’an, Sichuan | 30° 06' 49" N, 102° 48' 36" E | 799 | KP211434 | — |
| *B. maydis* | T21 | Group 1 | Maize | 2011 | Ya’an, Sichuan | 29° 57' 16" N, 103° 07' 13" E | 574 | KP211435 | — |
| *B. maydis* | T28 | Group 1 | Maize | 2011 | Ya’an, Sichuan | 29° 57' 16" N, 103° 07' 13" E | 574 | KP211436 | — |
| *B. maydis* | F1 | Group 1 | Maize | 2012 | Mianyang, Sichuan | 31° 40' 40" N, 104° 41' 11" E | 534 | KF922852 | KR424485 |
| *B. maydis* | F2 | Group 1 | Maize | 2012 | Luzhou, Sichuan | 29° 08' 12" N, 105° 24' 06" E | 320 | KF922853 | KR424486 |
| *B. maydis* | F3 | Group 1 | Maize | 2012 | Leshan, Sichuan | 29° 32' 14" N, 103° 44' 11" E | 362 | KF922854 | — |
| *B. maydis* | F4 | Group 1 | Maize | 2012 | Leshan, Sichuan | 29° 32' 14" N, 103° 44' 11" E | 362 | KF922855 | KR424487 |
| *B. maydis* | F5 | Group 1 | Maize | 2012 | Luzhou, Sichuan | 28° 07' 56" N, 106°04' 54" E | 889 | KF922856 | KR424488 |
| *B. maydis* | F6 | Group 1 | Maize | 2012 | Luzhou, Sichuan | 28° 07' 56" N, 106°04' 54" E | 889 | KF922857 | KR424489 |
| *B. maydis* | F7 | Group 1 | Maize | 2012 | Leshan, Sichuan | 29° 32' 14" N, 103° 44' 11" E | 362 | KF922858 | KR424490 |
| *B. maydis* | F13 | Group 1 | Maize | 2012 | Luzhou, Sichuan | 28° 07' 56" N, 106°04' 54" E | 889 | KF922859 | KR424493 |
| *B. maydis* | F17 | Group 1 | Maize | 2013 | Chengdu, Sichuan | 30° 46' 61" N, 104° 12' 27" E | 489 | KP211427 | KR424495 |
| *B. maydis* | F18 | Group 1 | Maize | 2013 | Chengdu, Sichuan | 30° 46' 61" N, 104° 12' 27" E | 489 | — | KR424496 |
| *B. maydis* | F20 | Group 1 | Maize | 2013 | Chengdu, Sichuan | 30° 46' 61" N, 104° 12' 27" E | 489 | KF922860 | KR424497 |
| *B. maydis* | F21 | Group 1 | Maize | 2012 | Chengdu, Sichuan | 30° 46' 61" N, 104° 12' 27" E | 489 | KF922861 | KR424498 |
| *B. maydis* | F22 | Group 1 | Maize | 2013 | Chengdu, Sichuan | 30° 46' 61" N, 104° 12' 27" E | 489 | KF922862 | KR424499 |
| *B. maydis* | F23 | Group 1 | Maize | 2012 | Chengdu, Sichuan | 30° 46' 61" N, 104° 12' 27" E | 489 | — | KR424500 |
| *B. maydis* | F25 | Group 1 | Maize | 2013 | Chengdu, Sichuan | 30° 46' 61" N, 104° 12' 27" E | 489 | KF922863 | KR424501 |
| *B. maydis* | F26 | Group 1 | Maize | 2012 | Leshan, Sichuan | 29° 32' 14" N, 103° 44' 11" E | 362 | KF922864 | KR424502 |
| *B. maydis* | F27 | Group 1 | Maize | 2013 | Mianyang, Sichuan | 31° 03' 29" N, 105° 06' 04" E | 370 | KF922865 | KR424503 |
| *B. maydis* | F28 | Group 1 | Maize | 2012 | Leshan, Sichuan | 29° 32' 14" N, 103° 44' 11" E | 362 | KF922866 | KR424504 |
| *B. maydis* | F29 | Group 1 | Maize | 2013 | Leshan, Sichuan | 29° 32' 14" N, 103° 44' 11" E | 362 | KF922867 | KR424505 |
| *B. maydis* | F31 | Group 1 | Maize | 2013 | Mianyang, Sichuan | 31° 22' 48" N, 104° 51' 23" E | 456 | KF922868 | KR424506 |
| *B. maydis* | F32 | Group 1 | Maize | 2013 | Mianyang, Sichuan | 31° 22' 48" N, 104° 51' 23" E | 456 | KP211428 | KR424507 |
| *B. maydis* | F33 | Group 1 | Maize | 2012 | Leshan, Sichuan | 29° 31' 28" N, 103° 59' 52" E | 384 | KF922869 | KR424508 |
| *B. maydis* | F36 | Group 1 | Maize | 2012 | Leshan, Sichuan | 29° 31' 28" N, 103° 59' 52" E | 384 | — | KR424509 |
| *B. maydis* | F37 | Group 1 | Maize | 2012 | Leshan, Sichuan | 29° 31' 28" N, 103° 59' 52" E | 384 | KF922870 | KR424510 |
| *B. maydis* | F38 | Group 1 | Maize | 2013 | Bazhong, Sichuan | 31° 45' 32" N, 106° 41' 14" E | 510 | KF922871 | KR424511 |
| *B. maydis* | F39 | Group 1 | Maize | 2012 | Yibin, Sichuan | 28° 33' 20" N, 104° 44' 42" E | 360 | — | KR424512 |
| *B. maydis* | F42 | Group 1 | Maize | 2013 | Chengdu, Sichuan | 30° 43' 14" N, 103° 52' 06" E | 537 | KP211429 | KR424513 |
| *B. maydis* | F46 | Group 1 | Maize | 2012 | Deyang, Sichuan | 31° 02' 43" N, 104° 18' 04" E | 484 | KF922872 | KR424514 |
| *B. maydis* | F47 | Group 1 | Maize | 2012 | Deyang, Sichuan | 31° 02' 43" N, 104° 18' 04" E | 484 | — | KR424515 |
| *B. maydis* | F48 | Group 1 | Maize | 2013 | Meishan, Sichuan | 30° 19' 07" N, 103° 52' 02" E | 427 | KF922874 | KR424516 |
| *B. maydis* | F49 | Group 1 | Maize | 2013 | Meishan, Sichuan | 30° 19' 07" N, 103° 52' 02" E | 427 | KF922875 | KR424517 |
| *B. maydis* | F50 | Group 1 | Maize | 2012 | Yibin, Sichuan | 28° 15' 58" N, 104° 41' 24" E | 376 | — | KR424518 |
| *B. maydis* | F51 | Group 1 | Maize | 2012 | Yibin, Sichuan | 28° 15' 58" N, 104° 41' 24" E | 376 | — | KR424519 |
| *B. maydis* | F52 | Group 1 | Maize | 2013 | Meishan, Sichuan | 30° 18' 28" N, 103° 51' 50" E | 442 | KP211430 | — |
| *B. maydis* | F53 | Group 1 | Maize | 2012 | Zigong, Sichuan | 29° 18' 10" N, 104° 31' 01" E | 328 | KP211431 | KR424520 |
| *B. maydis* | BM1 | Group 1 | Maize | 2015 | Deyang, Sichuan | 30° 57' 27" N, 104° 39' 52" E | 467 | KX834866 | KX835060 |
| *B. maydis* | BM2 | Group 1 | Maize | 2015 | Meishan, Sichuan | 29° 50' 08" N, 103° 25' 01" E | 466 | KX834867 | KX835061 |
| *B. maydis* | BM3 | Group 1 | Maize | 2015 | Deyang, Sichuan | 30° 57' 27" N, 104° 39' 52" E | 467 | KX834868 | KX835062 |
| *B. maydis* | BM4 | Group 1 | Maize | 2015 | Meishan, Sichuan | 29° 50' 08" N, 103° 25 '01" E | 466 | KX834869 | KX835063 |
| *B. maydis* | BM5 | Group 1 | Maize | 2015 | Chengdu, Sichuan | 30° 28' 51" N, 103° 53' 21" E | 466 | KX834870 | KX835064 |
| *B. maydis* | BM6 | Group 1 | Maize | 2015 | Mianyang, Sichuan | 31° 39' 10" N, 105° 09' 26" E | 468 | KX834871 | KX835065 |
| *B. maydis* | BM7 | Group 1 | Maize | 2015 | Chengdu, Sichuan | 30° 01' 28" N, 103° 24' 43" E | 561 | KX834872 | KX835066 |
| *B. maydis* | BM8 | Group 1 | Maize | 2015 | Deyang, Sichuan | 30° 57' 27" N, 104° 39' 52" E | 467 | KX834873 | KX835067 |
| *B. maydis* | BM9 | Group 1 | Maize | 2015 | Chengdu, Sichuan | 30° 22' 32" N, 103° 31' 37" E | 488 | KX834874 | KX835068 |
| *B. maydis* | BM10 | Group 1 | Maize | 2015 | Meishan, Sichuan | 30° 03' 56" N, 104° 13' 30" E | 386 | KX834875 | KX835069 |
| *B. maydis* | BM11 | Group 1 | Maize | 2015 | Meishan, Sichuan | 30° 03' 56" N, 104° 13' 30" E | 386 | KX834876 | KX835070 |
| *B. maydis* | BM12 | Group 1 | Maize | 2015 | Deyang, Sichuan | 30° 57' 27" N, 104° 39' 52" E | 467 | KX834877 | KX835071 |
| *B. maydis* | BM13 | Group 1 | Maize | 2015 | Meishan, Sichuan | 30° 03' 56" N, 104° 13' 30" E | 386 | KX834878 | KX835072 |
| *B. maydis* | BM14 | Group 1 | Maize | 2015 | Meishan, Sichuan | 30° 03' 56" N, 104° 13' 30" E | 386 | — | KX835073 |
| *B. maydis* | BM15 | Group 1 | Maize | 2015 | Deyang, Sichuan | 30° 57' 27" N, 104° 39' 52" E | 467 | KX834879 | KX835074 |
| *B. maydis* | BM16 | Group 1 | Maize | 2015 | Chengdu, Sichuan | 30° 54' 50" N, 103° 49' 15" E | 584 | KX834880 | KX835075 |
| *B. maydis* | BM17 | Group 1 | Maize | 2015 | Deyang, Sichuan | 30° 57' 27" N, 104° 39' 52" E | 467 | KX834881 | KX835076 |
| *B. maydis* | BM18 | Group 1 | Maize | 2015 | Chengdu, Sichuan | 30° 54' 50" N, 103° 49' 15" E | 584 | KX834882 | KX835077 |
| *B. maydis* | BM19 | Group 1 | Maize | 2015 | Deyang, Sichuan | 30° 56' 49" N, 104° 36' 16" E | 476 | KX834883 | KX835078 |
| *B. maydis* | BM20 | Group 1 | Maize | 2015 | Deyang, Sichuan | 30° 56' 49" N, 104° 36' 16" E | 476 | KX834884 | KX835079 |
| *B. maydis* | BM21 | Group 1 | Maize | 2015 | Meishan, Sichuan | 30° 04' 01" N, 104° 13' 25" E | 382 | KX834885 | KX835080 |
| *B. maydis* | BM22 | Group 1 | Maize | 2015 | Neijiang, Sichuan | 29° 30' 01" N, 104° 34' 06" E | 350 | KX834886 | KX835081 |
| *B. maydis* | BM23 | Group 1 | Maize | 2015 | Neijiang, Sichuan | 29° 30' 01" N, 104° 34' 06" E | 350 | KX834887 | KX835082 |
| *B. maydis* | BM24 | Group 1 | Maize | 2015 | Meishan, Sichuan | 30° 04' 01" N, 104° 13' 25" E | 382 | KX834888 | KX835083 |
| *B. maydis* | BM25 | Group 1 | Maize | 2015 | Meishan, Sichuan | 30° 04' 01" N, 104° 13' 25" E | 382 | KX834889 | KX835084 |
| *B. maydis* | BM26 | Group 1 | Maize | 2015 | Deyang, Sichuan | 30° 36' 59" N, 105° 01' 39" E | 450 | KX834890 | KX835085 |
| *B. maydis* | BM27 | Group 1 | Maize | 2015 | Deyang, Sichuan | 30° 36' 59" N, 105° 01' 39" E | 450 | KX834891 | KX835086 |
| *B. maydis* | BM28 | Group 1 | Maize | 2015 | Deyang, Sichuan | 30° 36' 59" N, 105° 01' 39" E | 450 | KX834892 | — |
| *B. maydis* | BM29 | Group 1 | Maize | 2015 | Deyang, Sichuan | 30° 36' 59" N, 105° 01' 39" E | 450 | KX834893 | KX835087 |
| *B. maydis* | BM30 | Group 1 | Maize | 2015 | Deyang, Sichuan | 30° 36' 59" N, 105° 01' 39" E | 450 | KX834894 | KX835088 |
| *B. maydis* | BM31 | Group 1 | Maize | 2015 | Meishan, Sichuan | 30° 03' 56" N, 104° 13' 30" E | 386 | KX834895 | KX835089 |
| *B. maydis* | BM32 | Group 1 | Maize | 2015 | Ya’an, Sichuan | 30° 24' 18" N, 102° 46' 20" E | 1197 | KX834896 | KX835090 |
| *B. maydis* | BM33 | Group 1 | Maize | 2015 | Deyang, Sichuan | 30° 36' 59" N, 105° 01' 39" E | 450 | KX834897 | KX835091 |
| *B. maydis* | BM34 | Group 1 | Maize | 2015 | Zigong, Sichuan | 29° 18' 01" N, 104° 34' 57" E | 314 | KX834898 | KX835092 |
| *B. maydis* | BM35 | Group 1 | Maize | 2015 | Meishan, Sichuan | 30° 03' 56" N, 104° 13' 30" E | 386 | KX834899 | KX835093 |
| *B. zeicola* | T3 | Group 2 | Maize | 2011 | Ya’an, Sichuan | 30° 24' 19" N, 102° 46' 21" E | 1049 | KP211440 | — |
| *B. zeicola* | T4 | Group 2 | Maize | 2011 | Ya’an, Sichuan | 30° 00' 16" N, 103° 05' 52" E | 620 | — | — |
| *B. zeicola* | T6 | Group 2 | Maize | 2011 | Ya’an, Sichuan | 29° 59' 59" N, 102° 50' 59" E | 665 | KP211441 | — |
| *B. zeicola* | T10 | Group 2 | Maize | 2011 | Ya’an, Sichuan | 30° 24' 11" N, 102° 46' 06" E | 1072 | KP211442 | — |
| *B. zeicola* | T11 | Group 2 | Maize | 2011 | Ya’an, Sichuan | 30° 29' 16" N, 102° 52' 34" E | 1420 | KP211443 | — |
| *B. zeicola* | T15 | Group 2 | Maize | 2011 | Ya’an, Sichuan | 29° 59' 45" N, 102° 51' 39" E | 653 | — | — |
| *B. zeicola* | T17 | Group 2 | Maize | 2011 | Ya’an, Sichuan | 30° 24' 11" N, 102° 46' 06" E | 1072 | KP211444 | — |
| *B. zeicola* | T18 | Group 2 | Maize | 2011 | Ya’an, Sichuan | 30° 29' 16" N, 102° 52' 34" E | 1420 | KP211445 | — |
| *B. zeicola* | T22 | Group 2 | Maize | 2011 | Ya’an, Sichuan | 30° 08' 19" N, 103° 02' 56" E | 908 | KP211446 | — |
| *B. zeicola* | T25 | Group 2 | Maize | 2011 | Ya’an, Sichuan | 29° 56' 22" N, 102° 55' 07" E | 678 | KP211447 | — |
| *B. zeicola* | T26 | Group 2 | Maize | 2011 | Ya’an, Sichuan | 29° 56' 22" N, 102° 55' 07" E | 678 | KP211448 | — |
| *B. zeicola* | T27 | Group 2 | Maize | 2011 | Mianyang, Sichuan | 31° 41' 58" N, 104° 27' 53" E | 657 | KP211449 | — |
| *B. zeicola* | F8 | Group 2 | Maize | 2012 | Yibin, Sichuan | 28° 15' 58" N, 104° 41' 24" E | 376 | — | KR424521 |
| *B. zeicola* | F9 | Group 2 | Maize | 2012 | Yibin, Sichuan | 28° 15' 58" N, 104° 41' 24" E | 376 | KP211437 | KR424522 |
| *B. zeicola* | F10 | Group 2 | Maize | 2012 | Luzhou, Sichuan | 29° 08' 12" N, 105° 24' 06" E | 320 | KF922876 | KR424523 |
| *B. zeicola* | F14 | Group 2 | Maize | 2012 | Ya’an, Sichuan | 30° 10' 37" N, 103° 04' 41" E | 934 | KF922877 | KR424524 |
| *B. zeicola* | F19 | Group 2 | Maize | 2013 | Chengdu, Sichuan | 30° 43' 14" N, 103° 52' 06" E | 537 | KF922878 | KR424526 |
| *B. zeicola* | F24 | Group 2 | Maize | 2013 | Ya’an, Sichuan | 30° 06' 04" N, 103° 0' 59" E | 967 | KF922879 | KR424527 |
| *B. zeicola* | F30 | Group 2 | Maize | 2012 | Lashan, Sichuan | 29° 32' 14" N, 103° 44' 11" E | 362 | KF922880 | KR424528 |
| *B. zeicola* | F34 | Group 2 | Maize | 2013 | Mianyang, Sichuan | 31° 46' 28" N, 104° 26' 40" E | 819 | KF922881 | — |
| *B. zeicola* | F35 | Group 2 | Maize | 2013 | Mianyang, Sichuan | 31° 46' 28" N, 104° 26' 40" E | 819 | KF922882 | KR424529 |
| *B. zeicola* | F40 | Group 2 | Maize | 2012 | Yibin, Sichuan | 28° 47' 36" N, 105° 04' 36" E | 453 | KP211438 | KR424530 |
| *B. zeicola* | F41 | Group 2 | Maize | 2013 | Chengdu, Sichuan | 30° 43' 14" N, 103° 52' 06" E | 537 | KP211439 | KR424531 |
| *B. zeicola* | F43 | Group 2 | Maize | 2012 | Xichang, Sichuan | 27° 35' 10" N, 102° 11' 55" E | 1566 | KF922883 | KR424532 |
| *B. zeicola* | F44 | Group 2 | Maize | 2012 | Meishan, Sichuan | 30° 0' 58" N, 103° 30' 10" E | 482 | KF922884 | KR424533 |
| *B. zeicola* | F45 | Group 2 | Maize | 2012 | Meishan, Sichuan | 30° 0' 58" N, 103° 30' 10" E | 482 | — | KR424534 |
| *B. zeicola* | B1 | Group 2 | Maize | 2014 | Ya’an, Sichuan | 30° 06' 21" N, 103° 01' 01" E | 1234 | KR424460 | KR424535 |
| *B. zeicola* | B3 | Group 2 | Maize | 2014 | Ya’an, Sichuan | 30° 06' 21" N, 103° 01' 01" E | 1234 | KR424461 | KR424536 |
| *B. zeicola* | B4 | Group 2 | Maize | 2014 | Ya’an, Sichuan | 30° 06' 21" N, 103° 01' 01" E | 1234 | KR424462 | KR424537 |
| *B. zeicola* | B7 | Group 2 | Maize | 2014 | Chengdu, Sichuan | 30° 43' 14" N, 103° 52' 06" E | 537 | KR424463 | KR424538 |
| *B. zeicola* | B8 | Group 2 | Maize | 2014 | Ya’an, Sichuan | 29° 57' 18" N, 103° 06' 46" E | 526 | KR424464 | KR424539 |
| *B. zeicola* | B9 | Group 2 | Maize | 2014 | Ya’an, Sichuan | 29° 57' 18" N, 103° 06' 46" E | 526 | KR424465 | KR424540 |
| *B. zeicola* | B10 | Group 2 | Maize | 2014 | Ya’an, Sichuan | 29° 57' 18" N, 103° 06' 46" E | 526 | KR424466 | KR424541 |
| *B. zeicola* | B11 | Group 2 | Maize | 2014 | Deyang, Sichuan | 31° 05' 07" N, 104° 16' 03" E | 451 | KR424467 | KR424542 |
| *B. zeicola* | B12 | Group 2 | Maize | 2014 | Ya’an, Sichuan | 29° 57' 52" N, 103° 08' 56" E | 549 | KR424468 | — |
| *B. zeicola* | B13 | Group 2 | Maize | 2014 | Ya’an, Sichuan | 29° 57' 52" N, 103° 08' 56" E | 549 | KR424469 | KR424543 |
| *B. zeicola* | B14 | Group 2 | Maize | 2014 | Chengdu, Sichuan | 30° 43' 14" N, 103° 52' 06" E | 537 | KR424470 | KR424544 |
| *B. zeicola* | B15 | Group 2 | Maize | 2014 | Meishan, Sichuan | 30° 04' 14" N, 104° 12' 55" E | 409 | KR424471 | KR424545 |
| *B. zeicola* | B16 | Group 2 | Maize | 2014 | Deyang, Sichuan | 31° 05' 07" N, 104° 16' 03" E | 451 | KR424472 | KR424546 |
| *B. zeicola* | B17 | Group 2 | Maize | 2014 | Deyang, Sichuan | 31° 05' 07" N, 104° 16' 03" E | 451 | KR424473 | KR424547 |
| *B. zeicola* | B18 | Group 2 | Maize | 2014 | Ya’an, Sichuan | 29° 59' 03" N, 102° 58' 56" E | 591 | KR424474 | KR424548 |
| *B. zeicola* | B19 | Group 2 | Maize | 2014 | Ya’an, Sichuan | 29° 59' 03" N, 102° 58' 56" E | 591 | KR424475 | KR424549 |
| *B. zeicola* | B20 | Group 2 | Maize | 2014 | Ya’an, Sichuan | 29° 59' 03" N, 102° 58' 56" E | 591 | KR424476 | KR424550 |
| *B. zeicola* | B22 | Group 2 | Maize | 2014 | Ya’an, Sichuan | 29° 59' 03" N, 102° 58' 56" E | 591 | KR424477 | KR424551 |
| *B. zeicola* | B23 | Group 2 | Maize | 2014 | Deyang, Sichuan | 31° 05' 07" N, 104° 16' 03" E | 451 | KR424478 | KR424552 |
| *B. zeicola* | B24 | Group 2 | Maize | 2014 | Ya’an, Sichuan | 29° 59' 28" N, 102° 58' 52" E | 619 | KR424479 | KR424553 |
| *B. zeicola* | B26 | Group 2 | Maize | 2014 | Ya’an, Sichuan | 29° 59' 28" N, 102° 58' 52" E | 619 | KR424480 | KR424554 |
| *B. zeicola* | B28 | Group 2 | Maize | 2014 | Ya’an, Sichuan | 29° 59' 28" N, 102° 58' 52" E | 619 | KR424481 | KR424555 |
| *B. zeicola* | B29 | Group 2 | Maize | 2014 | Ya’an, Sichuan | 29° 59' 28" N, 102° 58' 52" E | 619 | KR424482 | KR424556 |
| *B. zeicola* | B30 | Group 2 | Maize | 2014 | Ya’an, Sichuan | 29° 59' 28" N, 102° 58' 52" E | 619 | KR424483 | KR424557 |
| *B. zeicola* | B36 | Group 2 | Maize | 2014 | Ya’an, Sichuan | 29° 59' 28" N, 102° 58' 52" E | 619 | KR424484 | KR424558 |
| *B. zeicola* | BZ1 | Group 2 | Maize | 2015 | Chengdu, Sichuan | 30° 22' 32" N, 103° 31' 37" E | 488 | KX834901 | KX835095 |
| *B. zeicola* | BZ2 | Group 2 | Maize | 2015 | Chengdu, Sichuan | 30° 22' 32" N, 103° 31' 37" E | 488 | KX834902 | KX835096 |
| *B. zeicola* | BZ3 | Group 2 | Maize | 2015 | Chengdu, Sichuan | 30° 22' 32" N, 103° 31' 37" E | 488 | KX834903 | KX835097 |
| *B. zeicola* | BZ4 | Group 2 | Maize | 2015 | Chengdu, Sichuan | 30° 22' 32" N, 103° 31' 37" E | 488 | KX834904 | KX835098 |
| *B. zeicola* | BZ5 | Group 2 | Maize | 2015 | Deyang, Sichuan | 30° 57' 27" N, 104° 39' 52" E | 467 | KX834905 | KX835099 |
| *B. zeicola* | BZ6 | Group 2 | Maize | 2015 | Chengdu, Sichuan | 30° 22' 32" N, 103° 31' 37" E | 488 | KX834906 | KX835100 |
| *B. zeicola* | BZ7 | Group 2 | Maize | 2015 | Chengdu, Sichuan | 30° 22' 32" N, 103° 31' 37" E | 488 | KX834907 | KX835101 |
| *B. zeicola* | BZ8 | Group 2 | Maize | 2015 | Chengdu, Sichuan | 30° 22' 32" N, 103° 31' 37" E | 488 | KX834908 | KX835102 |
| *B. zeicola* | BZ9 | Group 2 | Maize | 2015 | Neijiang, Sichuan | 29° 30' 01" N, 104° 34' 06" E | 350 | KX834909 | KX835103 |
| *B. zeicola* | BZ10 | Group 2 | Maize | 2015 | Deyang, Sichuan | 30° 57' 27" N, 104° 39' 52" E | 467 | KX834910 | KX835104 |
| *B. zeicola* | BZ11 | Group 2 | Maize | 2015 | Deyang, Sichuan | 30° 57' 27" N, 104° 39' 52" E | 467 | KX834911 | KX835105 |
| *B. zeicola* | BZ12 | Group 2 | Maize | 2015 | Deyang, Sichuan | 30° 57' 27" N, 104° 39' 52" E | 467 | KX834912 | KX835106 |
| *B. zeicola* | BZ13 | Group 2 | Maize | 2015 | Deyang, Sichuan | 30° 57' 27" N, 104° 39' 52" E | 467 | KX834913 | KX835107 |
| *B. zeicola* | BZ14 | Group 2 | Maize | 2015 | Chengdu, Sichuan | 30° 46' 61" N, 104° 12' 27" E | 489 | — | KX835108 |
| *B. zeicola* | BZ15 | Group 2 | Maize | 2015 | Chengdu, Sichuan | 30° 46' 61" N, 104° 12' 27" E | 489 | KX834914 | KX835109 |
| *B. zeicola* | BZ16 | Group 2 | Maize | 2015 | Chengdu, Sichuan | 30° 46' 61" N, 104° 12' 27" E | 489 | KX834915 | KX835110 |
| *B. zeicola* | BZ17 | Group 2 | Maize | 2015 | Chengdu, Sichuan | 30° 46' 61" N, 104° 12' 27" E | 489 | KX834916 | KX835111 |
| *B. zeicola* | BZ18 | Group 2 | Maize | 2015 | Deyang, Sichuan | 30° 56' 49" N, 104° 36' 16" E | 476 | KX834917 | KX835112 |
| *B. zeicola* | BZ19 | Group 2 | Maize | 2015 | Chengdu, Sichuan | 30° 46' 61" N, 104° 12' 27" E | 489 | KX834918 | KX835113 |
| *B. zeicola* | BZ20 | Group 2 | Maize | 2015 | Deyang, Sichuan | 30° 56' 49" N, 104° 36' 16" E | 476 | KX834919 | KX835114 |
| *B. zeicola* | BZ21 | Group 2 | Maize | 2015 | Deyang, Sichuan | 30° 56' 49" N, 104° 36' 16" E | 476 | KX834920 | KX835115 |
| *B. zeicola* | BZ22 | Group 2 | Maize | 2015 | Deyang, Sichuan | 30° 36' 59" N, 105° 01' 39" E | 450 | KX834921 | KX835116 |
| *B. zeicola* | BZ23 | Group 2 | Maize | 2015 | Deyang, Sichuan | 30° 36' 59" N, 105° 01' 39" E | 450 | KX834922 | KX835117 |
| *B. zeicola* | BZ24 | Group 2 | Maize | 2015 | Chengdu, Sichuan | 30° 46' 61" N, 104° 12' 27" E | 489 | KX834923 | KX835118 |
| *B. zeicola* | BZ25 | Group 2 | Maize | 2015 | Meishan, Sichuan | 30° 03' 56" N, 104° 13' 30" E | 386 | KX834924 | KX835119 |
| *B. zeicola* | BZ26 | Group 2 | Maize | 2015 | Deyang, Sichuan | 30° 36' 59" N, 105° 01' 39" E | 450 | KX834925 | KX835120 |
| *B. zeicola* | BZ27 | Group 2 | Maize | 2015 | Deyang, Sichuan | 30° 36' 59" N, 105° 01' 39" E | 450 | KX834926 | KX835121 |
| *B. zeicola* | BZ28 | Group 2 | Maize | 2015 | Neijiang, Sichuan | 29° 30' 01" N, 104° 34' 06" E | 350 | KX834927 | KX835122 |
| *B. zeicola* | BZ29 | Group 2 | Maize | 2015 | Deyang, Sichuan | 30° 36' 59" N, 105° 01' 39" E | 450 | KX834928 | KX835123 |
| *B. zeicola* | BZ30 | Group 2 | Maize | 2014 | Leshan, Sichuan | 29° 28' 22" N, 104° 02' 39" E | 405 | KX834929 | KX835124 |
| *B. zeicola* | BZ31 | Group 2 | Maize | 2014 | Leshan, Sichuan | 29° 28' 22" N, 104° 02' 39" E | 405 | KX834930 | KX835125 |
| *B. zeicola* | BZ32 | Group 2 | Maize | 2014 | Leshan, Sichuan | 29° 28' 22" N, 104° 02' 39" E | 405 | KX834931 | KX835126 |
| *B. zeicola* | BZ33 | Group 2 | Maize | 2014 | Leshan, Sichuan | 29° 28' 22" N, 104° 02' 39" E | 405 | KX834932 | KX835127 |
| *B. zeicola* | BZ34 | Group 2 | Maize | 2014 | Luzhou, Sichuan | 27° 59' 09" N, 105° 30' 04" E | 933 | KX834933 | KX835128 |
| *B. zeicola* | BZ35 | Group 2 | Maize | 2014 | Luzhou, Sichuan | 27° 59' 09" N, 105° 30' 04" E | 933 | KX834934 | KX835129 |
| *B. zeicola* | BZ36 | Group 2 | Maize | 2014 | Mianyang, Sichuan | 31° 38' 18" N, 104° 40' 39" E | 520 | KX834935 | KX835130 |
| *B. zeicola* | BZ37 | Group 2 | Maize | 2014 | Chengdu, Sichuan | 30° 59' 43" N, 103° 41' 45" E | 677 | KX834936 | KX835131 |
| *B. zeicola* | BZ38 | Group 2 | Maize | 2014 | Chengdu, Sichuan | 30° 59' 43" N, 103° 41' 45" E | 690 | KX834937 | KX835132 |
| *B. zeicola* | BZ39 | Group 2 | Maize | 2014 | Chengdu, Sichuan | 30° 59' 43" N, 103° 41' 45" E | 690 | KX834938 | — |
| *B. zeicola* | BZ40 | Group 2 | Maize | 2015 | Chengdu, Sichuan | 30° 58' 16" N, 103° 45' 47" E | 653 | KX834939 | KX835133 |
| *B. zeicola* | BZ41 | Group 2 | Maize | 2015 | Luding, Sichuan | 29° 52' 27" N, 102° 12' 35" E | 1479 | KX834940 | — |
| *B. cynodontis* | BC1 | Group 3 | Maize | 2015 | Deyang, Sichuan | 30° 57' 27" N, 104° 39' 52" E | 467 | KX834941 | KX835134 |
| *B. cynodontis* | BC2 | Group 3 | Maize | 2015 | Deyang, Sichuan | 30° 57' 27" N, 104° 39' 52" E | 467 | KX834942 | KX835135 |
| *B. cynodontis* | BC3 | Group 3 | Maize | 2015 | Deyang, Sichuan | 30° 57' 27" N, 104° 39' 52" E | 467 | KX834943 | KX835136 |
| *B. oryzae* | BO1 | Group 4 | Maize | 2015 | Neijiang, Sichuan | 29° 30' 01" N, 104° 34' 06" E | 350 | KX834944 | KX835137 |
| *B. setariae* | BS14 | Group 5 | Maize | 2015 | Chengdu, Sichuan | 30° 46' 61" N, 104° 12' 27" E | 489 | MG780266 | MK558813 |
| *B. setariae* | BS15 | Group 5 | Maize | 2015 | Chengdu, Sichuan | 30° 46' 61" N, 104° 12' 27" E | 489 | MG780265 | MK558814 |
| *B. saccharicola* | BM36 | Group 6 | Maize | 2015 | Meishan, Sichuan | 30° 03' 56" N, 104° 13' 30" E | 386 | KX834900 | KX835094 |

Notes: —, not available.
