# Supplementary material for: Etiology and Symptoms of Maize Leaf Spot Caused by Bipolaris spp. in Sichuan, China
Source: Pathogens. 2020 Mar 20;9(3):229. doi: 10.3390/pathogens9030229 (PMC7157660; doi:10.3390/pathogens9030229)
Supplement: Supplementary file 1 [file pathogens-09-00229-s001.zip › Supplementary files/Table S2. Symptoms and the virulence levels of Bipolaris isolates on maize.docx]

**Table S2.** Symptoms and the virulence levels of *Bipolaris* isolates on maize.

| **Isolates** | **Species** | **Field symptom types** | **Symptom of pathogenicity** | **Lesion length (mm)** | **Virulence level** |
| --- | --- | --- | --- | --- | --- |
| F50 | *B. maydis* | Type Ⅱ | elliptic | 8.0±0.57 | High |
| BM9 | *B. maydis* | Type I | elliptic | 7.0±0.63 | High |
| BM33 | *B. maydis* | Type Ⅱ | elliptic | 7.0±0.40 | High |
| F6 | *B. maydis* | Type Ⅱ | fusiform | 6.8±0.87 | High |
| BM27 | *B. maydis* | Type I | elliptic | 6.8±0.70 | High |
| BM2 | *B. maydis* | Type Ⅳ | fusiform | 6.8±0.55 | High |
| BM32 | *B. maydis* | Type I | fusiform | 6.6±0.82 | High |
| F4 | *B. maydis* | Type Ⅱ | fusiform | 6.6±0.56 | High |
| F48 | *B. maydis* | Type Ⅱ | subrotund | 6.6±0.74 | High |
| BM28 | *B. maydis* | Type Ⅱ | fusiform | 6.5±0.77 | High |
| BM23 | *B. maydis* | Type Ⅳ | subrotund | 6.3±0.65 | High |
| F2 | *B. maydis* | Type I | fusiform | 6.2±0.25 | High |
| F3 | *B. maydis* | Type Ⅳ | subrotund | 6.1±0.68 | High |
| F51 | *B. maydis* | Type I | fusiform | 6.1±0.36 | High |
| BM21 | *B. maydis* | Type I | elliptic | 6.1±0.87 | High |
| BM22 | *B. maydis* | Type Ⅴ | subrotund | 6.1±0.39 | High |
| BM29 | *B. maydis* | Type Ⅳ | subrotund | 6.0±0.63 | High |
| F31 | *B. maydis* | Type Ⅳ | subrotund | 5.2±0.72 | Moderate |
| F5 | *B. maydis* | Type I | elliptic | 5.2±0.37 | Moderate |
| BM1 | *B. maydis* | Type I | elliptic | 5.0±0.41 | Moderate |
| BM24 | *B. maydis* | Type I | subrotund | 5.0±0.27 | Moderate |
| F52 | *B. maydis* | Type Ⅱ | subrotund | 5.0±0.30 | Moderate |
| F1 | *B. maydis* | Type I | elliptic | 4.7±0.51 | Moderate |
| F7 | *B. maydis* | Type Ⅳ | subrotund | 4.6±0.26 | Moderate |
| F17 | *B. maydis* | Type Ⅳ | subrotund | 4.5±0.19 | Moderate |
| F37 | *B. maydis* | Type Ⅴ | elliptic | 4.5±0.57 | Moderate |
| F23 | *B. maydis* | Type I | subrotund | 4.3±0.21 | Moderate |
| F13 | *B. maydis* | Type I | subrotund | 4.3±0.23 | Moderate |
| F18 | *B. maydis* | Type Ⅴ | subrotund | 3.8±0.29 | Weak |
| BM15 | *B. maydis* | Type Ⅱ | subrotund | 3.5±0.38 | Weak |
| F53 | *B. maydis* | Type Ⅳ | subrotund | 3.2±0.22 | Weak |
| BM7 | *B. maydis* | Type I | subrotund | 3.2±0.70 | Weak |
| BM3 | *B. maydis* | Type I | subrotund | 3.1±0.59 | Weak |
| BM4 | *B. maydis* | Type Ⅳ | subrotund | 2.8±0.29 | Weak |
| F49 | *B. maydis* | Type Ⅱ | subrotund | 2.8±0.69 | Weak |
| BM5 | *B. maydis* | Type I | subrotund | 2.7±0.18 | Weak |
| BZ31 | *B. zeicola* | Type Ⅲ | narrow linear | 8.9±0.13 | High |
| B3 | *B. zeicola* | Type Ⅴ | narrow linear | 8.7±0.29 | High |
| B4 | *B. zeicola* | Type Ⅲ | narrow linear | 8.1±0.56 | High |
| B1 | *B. zeicola* | Type Ⅲ | narrow linear | 7.5±0.40 | High |
| BZ2 | *B. zeicola* | Type Ⅴ | narrow linear | 7.4±0.17 | High |
| BZ30 | *B. zeicola* | Type Ⅲ | narrow linear | 6.3±0.13 | High |
| F14 | *B. zeicola* | Type Ⅴ | narrow linear | 6.2±0.34 | High |
| F9 | *B. zeicola* | Type Ⅴ | subrotund | 5.1±0.13 | Moderate |
| BZ29 | *B. zeicola* | Type Ⅳ | subrotund | 4.5±0.18 | Moderate |
| BZ8 | *B. zeicola* | Type Ⅲ | subrotund | 4.5±0.44 | Moderate |
| B24 | *B. zeicola* | Type Ⅲ | subrotund | 4.4±0.31 | Moderate |
| BZ5 | *B. zeicola* | Type Ⅲ | subrotund | 4.4±0.22 | Moderate |
| BZ28 | *B. zeicola* | Type Ⅳ | subrotund | 4.4±0.27 | Moderate |
| F8 | *B. zeicola* | Type Ⅳ | subrotund | 4.1±0.10 | Moderate |
| F44 | *B. zeicola* | Type Ⅲ | subrotund | 4.0±0.14 | Moderate |
| B26 | *B. zeicola* | Type Ⅲ | subrotund | 4.0±0.13 | Moderate |
| B28 | *B. zeicola* | Type Ⅴ | subrotund | 4.0±0.23 | Moderate |
| B29 | *B. zeicola* | Type Ⅴ | subrotund | 3.9±0.42 | Weak |
| B7 | *B. zeicola* | Type Ⅲ | punctiform | 2.9±0.53 | Weak |
| BZ6 | *B. zeicola* | Type Ⅴ | punctiform | 2.9±0.43 | Weak |
| B30 | *B. zeicola* | Type Ⅲ | punctiform | 2.9±0.43 | Weak |
| B11 | *B. zeicola* | Type Ⅳ | punctiform | 2.9±0.38 | Weak |
| BZ4 | *B. zeicola* | Type Ⅳ | punctiform | 2.9±0.24 | Weak |
| BZ7 | *B. zeicola* | Type Ⅴ | punctiform | 2.6±0.31 | Weak |
| B36 | *B. zeicola* | Type Ⅴ | punctiform | 2.6±0.45 | Weak |
| F40 | *B. zeicola* | Type Ⅳ | punctiform | 2.6±0.24 | Weak |
| BZ9 | *B. zeicola* | Type Ⅲ | punctiform | 2.4±0.20 | Weak |
| BZ3 | *B. zeicola* | Type Ⅳ | punctiform | 2.3±0.28 | Weak |
| BZ1 | *B. zeicola* | Type Ⅳ | punctiform | 2.3±0.29 | Weak |
| F10 | *B. zeicola* | Type Ⅴ | punctiform | 2.1±0.16 | Weak |
| BZ10 | *B. zeicola* | Type Ⅳ | punctiform | 1.8±0.29 | Weak |
| BC1 | *B. cynodontis* | Type Ⅴ | punctiform | 2.0±0.20 | Weak |
| BC2 | *B. cynodontis* | Type Ⅴ | punctiform | 1.7±0.28 | Weak |
| BC3 | *B. cynodontis* | Type Ⅴ | punctiform | 1.8±0.19 | Weak |
| BO1 | *B. oryzae* | Type Ⅴ | punctiform | 1.9±0.36 | Weak |
| BS14 | *B. setariae* | Type Ⅳ | subrotund | 1.9±0.28 | Weak |
| BS15 | *B. setariae* | Type Ⅳ | subrotund | 2.3±0.10 | Weak |
| BM36 | *B. saccharicola* | Type Ⅳ | subrotund | 4.8±0.12 | Moderate |
