# Supplementary figures and images for: Etiology and Symptoms of Maize Leaf Spot Caused by Bipolaris spp. in Sichuan, China
Source: Pathogens. 2020 Mar 20;9(3):229. doi: 10.3390/pathogens9030229 (PMC7157660; doi:10.3390/pathogens9030229)

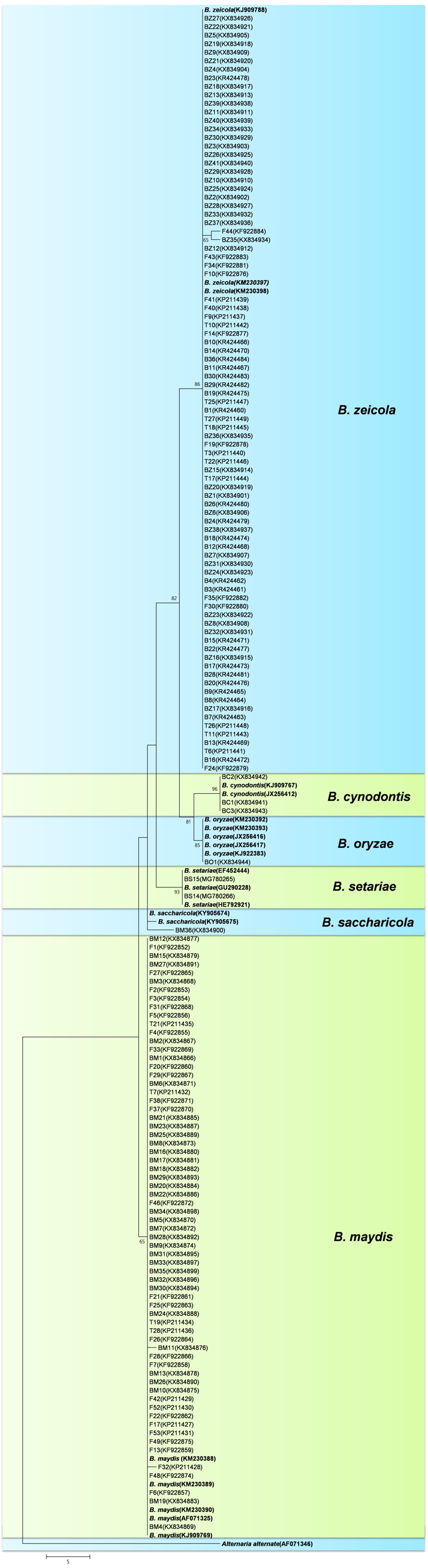

Supplement: Supplementary file 1 [file pathogens-09-00229-s001.zip › Supplementary files/Figure S2.jpg]

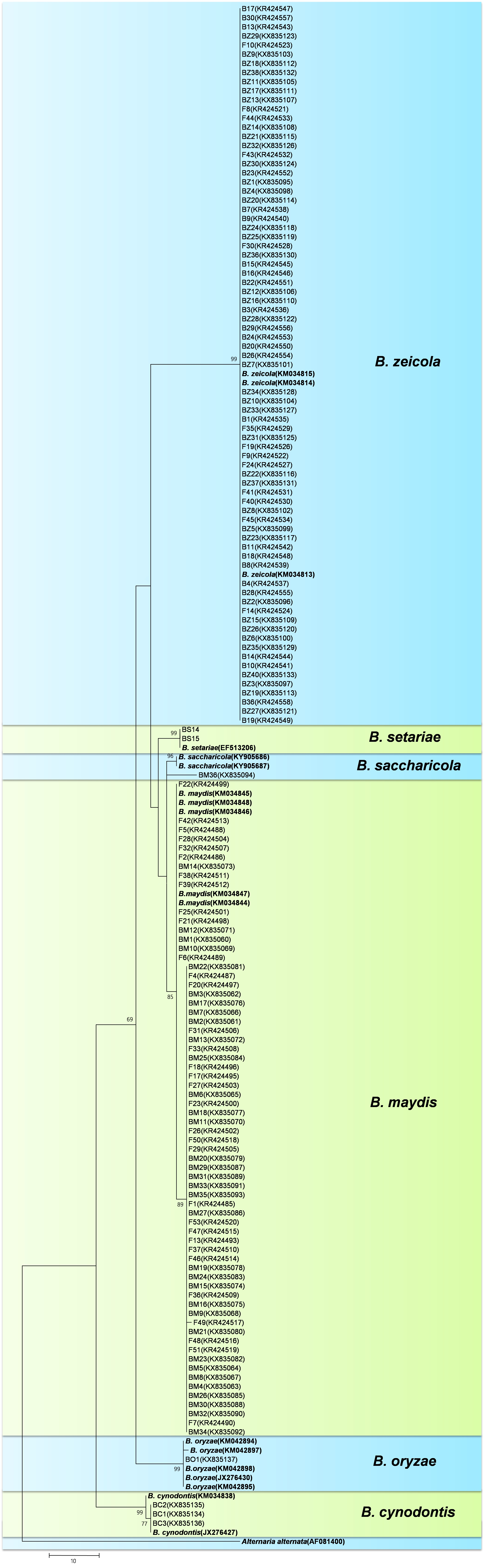

Supplement: Supplementary file 1 [file pathogens-09-00229-s001.zip › Supplementary files/Figure S3.jpg]
